# A systematic review to identify the use of stated preference research in the field of older adult care

**DOI:** 10.1007/s10433-022-00738-7

**Published:** 2022-11-07

**Authors:** Lea de Jong, Jan Zeidler, Kathrin Damm

**Affiliations:** Leibniz University Hannover, Center for Health Economics Research Hannover (CHERH), Otto-Brenner-Str.7, 30159 Hannover, Germany

**Keywords:** Stated preference, Systematic review, Elderly care, Older adult care, Long-term care, Preferences

## Abstract

In the design of long-term care systems, preferences can serve as an essential indication to better tailor services to the needs, wishes and expectations of its consumers. The aim of this systematic review was to summarize and synthesize available evidence on long-term care preferences that have been elicited by quantitative stated-preference methods. The databases PubMed and Web of Science were searched for the period 2000 to 2020 with an extensive set of search terms. Two independent researchers judged the eligibility of studies. The final number of included studies was 66, conducted in 19 different countries. Studies were systematized according to their content focus as well as the survey method used. Irrespective of the heterogeneity of studies with respect to research focus, study population, sample size and study design, some consistent findings emerged. When presented with a set of long-term care options, the majority of study participants preferred to “age in place” and make use of informal or home-based care. With increasing severity of physical and cognitive impairments, preferences shifted toward the exclusive use of formal care. Next to the severity of care needs, the influence on preferences of a range of other independent variables such as income, family status and education were tested; however, none showed consistent effects across all studies. The inclusion of choice-based elicitation techniques provides an impression of how studies operationalized long-term care and measured preferences. Future research should investigate how preferences might change over time and generations as well as people’s willingness and realistic capabilities of providing care.

## Introduction

Population aging, especially in developed countries, has long been recognized as a global phenomenon, with varied magnitude and projections worldwide. With the increasing population of older adults, substantial pressures are placed on national health and social care systems to adequately prepare for future challenges. The population of older adults aged 80 years or above is predicted to rise globally from 137 million in 2017 to 425 million in 2050 (United Nations, Department of Economic and Social Affairs, Population Division 2017). As an increase in age often coincides with an increasing likelihood of becoming care dependent, one major challenge that emerges is the delivery and financing of long-term care (LTC). In Europe, expenditure on LTC is expected to increase by 80% from 2015 to 2060 (Global Coalition on Aging 2018). According to the latest calculations of 2019, population aging will lead to an increase in the number of consumers of LTC services (United Nations, Department of Economic and Social Affairs, Population Division 2019).

When a person becomes care-dependent and requires support from others, that person can generally either receive care in a home-based, community, or institutional setting. In a home-based setting, in most instances, one or more informal caregivers are actively involved in providing care. Formal caregiving provided by a professional service often complements informal caregiving in a home-based setting, however, formal caregivers could also be the sole care-provider. The proportion of informal to formal caregiving varies internationally. This can be partly explained by the manner in which LTC systems are organized, which differ substantially across countries with regard to their funding sources, entitlements, service providers, access, LTC workforce, and quality control measures (Royal Commission into Aged Care Quality and Safety 2020). Additional influencing factors might be diverging views on the responsibility for providing care (family vs. government), different normative beliefs and perceived barriers to caregiving as well as individual willingness to provide care (Hoefman et al. 2017). Other societal changes, such as increasing employment rates of women, larger geographical distances between family members, and a growing number of single-person households, might also play an important role (Broese van Groenou and de Boer 2016). Such societal changes, alongside demographic developments and a rising demand for high-quality care, increase the pressure for national governments to act and establish sustainable and affordable LTC systems (Royal Commission into Aged Care Quality and Safety 2020).

In the designing of LTC systems, preferences can serve as an important indication to better tailor services to the needs and wishes of its consumers. Recently, there has been an increase in the involvement of patients and the general public in healthcare decision-making (Coulter 2012; Litva et al. 2002). In systems with limited resources, it is important to know which aspects of LTC are most and least important to people. Integrating people’s preferences in the design of services and products has also been linked to improved quality of care, quality of life, and overall well-being (Swift and Callahan 2009). Stated preference (SP) methods are used to ask participants directly about their preferences. Quantitative techniques can be used to infer people’s preferences by measuring a change in their utility function. As people are asked to trade-off between different aspects of care, it is possible to generate a ranking of said preferences. Examples of SP methods are contingent valuation (CV), best–worst scaling (BWS), discrete choice experiment (DCE), and other ranking or rating techniques (Klose et al. 2016). In the field of older adult care, SP methods have been increasingly applied since the last 10 to 15 years.

Preferences in the field of older adult care are multifaceted. It depends on multiple factors such as the study population, country in question, and the study’s perspective and focus. The broad thematic use of SP studies in the field of older adult care is mainly motivated by the complexity of LTC. Therefore, some studies have investigated preferences for specific LTC services (e.g., home-based care packages) in order to make preference-based suggestions for the improvement of LTC service designs (Lehnert et al. 2018; Chester et al. 2017, Kampanellou et al. 2019). Thereby, LTC services are decomposed into a set of attributes in order to measure underlying preferences with the help of the choices made by the respondents. Other studies used instruments to measure caregiver’s outcomes or investigate the suitability of different forms of LTC, providing an indirect measure of what LTC should look like according to people’s preferences (Al-Janabi et al. 2011; Milte et al. 2018a). In a recent scoping review by Lehnert et al. (2019), stated preferences for LTC were reviewed and summarized. It identified 12 qualitative, 40 quantitative, and seven mixed-methods studies in the field from a database search in February 2016. This systematic review builds upon the scoping review by additionally capturing the period from February 2016 to October 2020 and including preferences for nursing home as well as dementia care. The aim of this review is to summarize and synthesize available evidence on LTC preferences that have been elicited by quantitative SP methods.

## Methods

A systematic literature review was performed to identify original SP studies in the field of older adult care. Among others, these studies measure people’s willingness to provide care or their preferences for different LTC services (informal or formal). The systematic review was performed in accordance with the Preferred Reporting Items for Systematic Reviews and Meta-Analysis (PRISMA) guidelines (Moher et al. 2009).

### Search strategy and information sources

The literature search was conducted in September 2019 and updated in October 2020 using the scientific databases, PubMed and Web of Science. The search strategy combined English terms for older adult care [*care OR nursing OR elderly care OR long-term care OR LTC OR home care OR older adult care*] in search block A with search terms for SP elicitation methods [*stated preference(s) OR time trade-off OR TTO OR standard gamble OR conjoint OR contingent valuation OR discrete choice OR DCE OR willingness-to-pay OR WTP OR analytic hierarchy process OR AHP OR choice model OR best–worst scaling OR BWS OR willingness-to-accept OR WTA OR multi-criteria decision analysis OR MCDA OR multi-attribute utility OR MAUT*] in search block B. Search terms for block B were selected with the help of the literature survey on methods to perform systematic reviews of patient preferences by Yu et al. (2017). The Boolean operator “AND” combined the search terms of block A and B. Database-specific adjustments were made in block A in Web of Science by leaving out the search terms “care” and “nursing,” as these yielded a very high number of unspecific search hits in the database. Only one database-specific search filter was used in PubMed, specifically limiting the species to only humans. The timeframe was set to exclude studies published prior to 2000.

### Eligibility criteria and study selection

The selection process was based on a set of pre-defined inclusion and exclusion criteria. Studies were deemed eligible for inclusion if they (1) reported peer-reviewed, original quantitative data using SP methods such as DCE, BWS, CV, or other ranking or rating techniques, (2) were published in English or German, (3) were published in or after the year 2000, and (4) were focused specifically on LTC preferences of older adults in need of care. Studies were excluded if they (1) did not report original, peer-reviewed data (such as poster sessions, book chapters, reviews, reports and letter to the editors), (2) used qualitative or revealed preference methods to elicit preferences, (3) were published prior to 2000, or (4) focused on illness-related care, end-of-life care, or telecare. Studies focusing on specific illnesses or palliative care were excluded because of their tailored care needs and medical interventions that differ from general older adult care. Care for common cognitive and physical signs of old age such as incontinence, memory loss, and dementia were exempted from illness-related care and therefore included in this review. Based on these criteria, two independent researchers (de Jong, Damm) made a first selection by screening the titles and abstracts. Following the first selection, full texts of the remaining studies were screened and judged for inclusion. In case of disagreement, a third reviewer was consulted. Reference lists of included studies were hand-searched by the lead author.

### Data extraction and quality appraisal

Key characteristics of each study were extracted and collected in an extensive Microsoft Excel file. The extracted characteristics included the year of publication, country, aim of the study, characteristics of the study participants, sample size, instrument design including the software used, attributes and levels if applicable, data analysis, and key findings. A summary of key characteristics is presented in Table 1 and a detailed overview of selected characteristics is presented in Tables 2, 3, and 4. Tables 3, 4 and 5 additionally include the content-related assignment of each study into one of the four main entities (or “blocks”) identified below. The quality of this systematic review was ensured using the PRISMA 2009 Checklist (Moher et al. 2009). The quality of the included studies was assessed using the PREFS (*Purpose, Respondents, Explanation, Findings, Significance*) checklist (Joy et al. 2013). According to the PREFS checklist, studies are ranked on a scale from zero to five, with five indicating the highest methodological quality. Two researchers (de Jong, Damm) independently judged the quality of the studies. Table 5 presents an overview of the quality scores of the included studies.

## Results

**Fig. 1:**
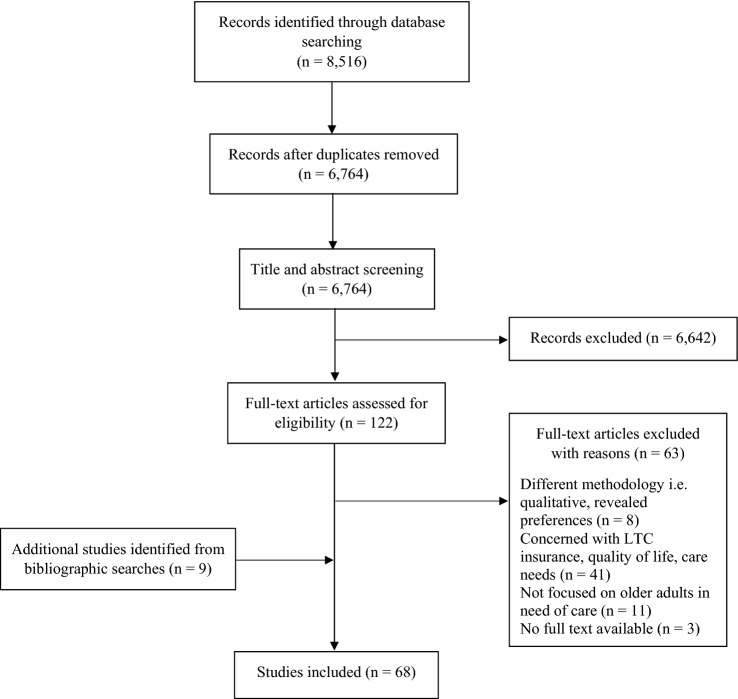
Flow diagram

In total, the search strategy yielded 8,516 articles to be screened for eligibility. After the removal of duplicates, two independent researchers screened and assessed 6,764 titles and abstracts with a set of predefined inclusion and exclusion criteria. As a result, 122 remaining full texts were assessed, of which 59 were ultimately included. Full texts with a different methodological or thematic focus, for instance, care needs or preferences for LTC insurance coverage were excluded. The reference lists of the included studies were additionally screened, which yielded another nine studies to be included. In total, 68 studies were included. The study selection process is shown in Fig. 1.

### Results of the quality appraisal

The results of the quality assessment are presented in Table 1. According to the checklist, most studies were deemed to have a good or very good quality. Three studies adequately addressed all five elements of the PREFS Checklist, and a further 46 addressed four out of five elements. Most often, studies did not report any evidence on differences between responders and non-responders of the sample, thereby neglecting to adequately assess potential selection bias. As preference studies often do not possess information on non-responders, other reviews have decided to disregard this area of the checklist from the appraisals (Lepper et al. 2020). Seventeen additional studies adequately reported three out of five elements of the checklist. Two studies adequately reported on less than three elements and were therefore excluded from the final sample (Chan et al. 2000, Li *and* Wang 2016). The results of the remaining 66 studies are described in the following sections.

**Table 1:** Summary of study characteristics (*n* = 66)

	n (%)	References
*Published year*		
2000 – 2002	5 (8)	(1–5)
2003 – 2005	6 (9)	(7–12)
2006 – 2008	5 (8)	(13–17)
2009 – 2011	8 (12)	(18–25)
2012 – 2014	4 (6)	(26–29)
2015 – 2017	7 (11)	(30–32, 34–37)
2017 – 2019	25 (38)	(38–62)
2020	6 (9)	(63–68)
*Country*		
Australia	3 (5)	(30, 43, 44)
China	16 (24)	(20, 25, 45–48, 56–60, 62–64, 67, 68)
France	1 (2)	(26)
Germany	3 (5)	(28, 42, 54)
Ireland	1 (2)	(66)
Israel	1 (2)	(35)
Korea	2 (3)	(8, 16)
Netherlands	1 (2)	(24)
United Kingdom	7 (11)	(21, 23, 31, 38, 39, 41, 52)
United States of America	18 (27)	(2, 3, 5, 7, 9–11, 13, 14, 17, 19, 27, 29, 36, 37, 49, 53, 61)
Philippines	1 (2)	(40)
Poland	1 (2)	(51)
Switzerland	3 (5)	(1, 55, 65)
Slovenia	1 (2)	(50)
Spain	2 (3)	(18, 34)
Taiwan	2 (3)	(12, 15)
Japan	1 (2)	(32)
Multiple Countries	2 (3)	(4, 22)
*Study Population*		
Care-dependent older adults	8 (12)	(5, 8, 17, 40, 45, 49, 53, 61)
Informal caregivers or family members as proxy	8 (12)	(2, 21–23, 26, 38, 41, 63)
Older hospital patients *(*> *60 years of age)*	2 (3)	(31, 39)
General population (*most often subpopulation: older adults* > *65 years*)	38 (58)	(3, 4, 7, 9–11, 13–15, 18, 19, 24, 25, 28, 32, 34–37, 42, 46–48, 50, 51, 54–60, 62, 64–68)
Multiple groups of study participants	10 (15)	(1, 12, 16, 20, 27, 29, 30, 43, 44, 52)
*Sample size*		
< 100	5 (8)	(13, 31, 37, 38, 62)
Between 100 and < 300	17 (26)	(1, 2, 9, 19, 21, 23, 26, 29, 30, 39–41, 43, 51–53, 64)
Between 300 and < 500	10 (15)	(10, 11, 14, 17, 27, 32, 35, 36, 63, 47)
Between 500 and < 1000	11 (17)	(15, 18, 20, 22, 43, 45, 46, 50, 57, 59, 66)
Between 1000 and < 2000	14 (21)	(4, 5, 7, 8, 12, 16, 24, 28, 42, 48, 54, 60, 61, 68)
≥ 2000	9 (14)	(3, 25, 34, 49, 55, 56, 58, 65, 67)
*Method*		
Best–worst scaling	1 (2)	(21)
Contingent valuation	6 (9)	(1, 22, 26, 27, 45, 63)
Conjoint analysis	3 (5)	(31, 39, 40)
Discrete choice experiment	12 (18)	(23, 24, 30, 32, 38, 41–44, 52, 64, 66)
Time-trade off	1 (2)	(37)
Vignette	11 (17)	(3, 5, 9, 10, 17, 19, 29, 35, 55, 61, 65)
Ranking	1 (2)	(62)
Rating	9 (14)	(2, 7, 13, 20, 36, 49, 50, 53, 54)
Questions with pre-specified categories (single choice, binary choice)	22 (33)	(4, 8, 11, 12, 14–16, 18, 25, 28, 34, 46–48, 51, 56–60, 67, 68)
*Administration of survey*		
Postal/Paper (*self-administered*)	14 (21)	(2, 4, 13, 14, 20, 21, 23, 32, 36, 49, 51, 55, 58, 65)
Online (*self-administered*)	3 (5)	(18, 24, 66)
Face-to-face or phone interviews (*participants were able to ask questions*)	44 (67)	(1, 3, 5, 7–12, 15–17, 19, 22, 25–28, 30, 31, 34, 35, 37–40, 43–48, 50, 53, 54, 56, 57, 59–63, 67, 68)
Multiple techniques	5 (8)	(29, 41, 42, 52, 64)

### Description of included studies

The included studies were published between the year 2000 and 2020 and the majority were conducted in China (*n* = 16) and the USA (*n *= 18). Two studies collected data from multiple countries, particularly from the UK, Spain, USA, and Sweden (Gustavsson et al. 2010) and Germany and the USA (Pinquart and Sörensen 2002). In total, studies conducted in 19 different countries were included. An overview of study characteristics is shown in Table 1. Barring two studies that investigated preferences over time (Wolff et al. 2008; Abbott et al. 2018), the remaining used a cross-sectional design. The sample size ranged from 28 (Chester et al. 2017) to 244,718 respondents (Roberts and Saliba 2019). The share of women in the sample ranged from 38.2% (Qian et al. 2017) to 100% (Kasper et al. 2000; Wolff et al. 2008). The majority of the included studies (*n* = 38) surveyed a sample of the general population, mostly older adults aged 65 years and above. Eight studies included care-dependent older adults and eight informal caregivers or family members. Ten studies used multiple groups of study participants for their analysis, specifically, care-dependent older adults as well as their primary caregivers or family members as proxy. Most often (*n* = 44) the surveys were administered via telephone or face-to-face interviews to enable participants to ask questions. Next to descriptive statistics, most studies additionally used inferential statistics to analyze their findings. Multinomial logistic regression, conditional logistic regression, and mixed logit models were most frequently used.

**Table 2:** Included studies using other stated preference techniques (*n* = 32)

Study^1^	Country	Population	Study objective	Method, type of measurement	Main results
(53) Block 3	USA	255 nursing home residents	To examine the change in nursing home residents’ importance ratings of everyday living preferences over time	**Rating** 72 preferences from 5 domains: 1. Self-dominion/autonomy in care, 2. Enlisting others in care, 3. Leisure and diversionary activities, 4. Social contact, 5. Growth activities	Most important preferences (%): 1) Have staff show you respect (96.50%); 2) Take care of your personal belongings or things (95.70%); 3) Have staff show they care about you (95.20%); 4) Have regular contact with family (93.30%); 5) Do what helps you feel better when you are upset (93.20%); 6) Choose who you would like involved in discussions about your care (92.90%)
(11) Block 1	USA	252 elders (age 55 and older) and 74 middle generation children of Great Lakes American Indians living in 3 residential areas	To explore differences in preferences between parent and middle generations for where and how LTC might be provided under conditions of dependency	**Single question with pre-specified categories** DV: “If you (your parent) could no longer take care of yourself (himself or herself) without help, which housing option would you prefer?” 1) Stay in own home with paid helpers, 2) Stay in own home with family help, 3) Move in with family, 4) Assisted living/foster care/group home, 5) Nursing home IV: age, education, sex, place of residence, index of illnesses, traditional culture, behaviors and attitudes related to LTC planning, language fluency	Preferred LTC option (Parent generation): 1) Stay in own home with paid helpers (30.4%), 2) Stay in own home with family help (31.3%), 3) Move in with family (4.4%), 4) Assisted living/foster care/group home (22.5%), 5) Nursing home (8.0%) Preferred LTC option (Middle generation): 1) Stay in own home with paid helpers (13.4%), 2) Stay in own home with family help (46.3%), 3) Move in with family (31.3%), 4) Assisted living/foster care/group home (5.9%), 5) Nursing home (3.1%) Preference for home care, Elder cohort*: female (OR: 2.08), traditional culture (OR: 1.25)
(15) Block 1	Taiwan	562 elderly people aged ≥ 65 years from 7 counties/cities in northern Taiwan	To investigate the preferences of the elderly in northern Taiwan with regard to various types of LTC services	**Single question with pre-specified categories** DV: “If you needed LTC services, taking every factor into practical consideration, which kind of LTC would you feel more inclined to accept?” 1) Institutional care, 2) Community-based care, 3) Home care IV: predisposing factors (age, gender, ethnic origin, marital status, education level, religion, living arrangements, economic condition), need factors (health condition, access to medical services, perceived health status, chronic diseases, outpatient visits, emergency visits, hospitalization history, regular physical examinations, preferred medical and rehabilitation services), subject’s understanding of and attitudes toward available LTC services	Preferred type of LTC: (1) Institutional care (16.72%), (2) Community-based care (9.61%), 3) Home care (73.67%) Home care vs. community-based care*: Visiting medical care team (OR: 2.12), Self-care information and others (OR: 4.39) Home care vs. institutional care*: Visiting medical care team (OR: 0.45)
(25) Block 1	China	20,255 older adults	To examine the willingness among older Chinese to live in eldercare institutions; to investigate the social, cultural, health and economic factors associated with such willingness	**Binary choice** (yes/no) DV: “Are you willing to live in an eldercare institution, such as an institution for respecting older adults, social welfare institution or senior apartment?” IV: predisposing factors (age, gender, education, marital status, having children, current living arrangement, willingness to live with children, family relations, socio-cultural beliefs and practices, psychological factors, knowledge and opinions), enabling factors (presence or local availability of eldercare institutions, monthly pension, savings for old age, self-assessed economic status), need factors (ADL, IADL, number of bed-ridden days, self-rated health status)	19.7% of urban respondents were willing to enter a care home compared to 16.6% of rural older Chinese. Factors influencing such willingness*: 1) Urban: age (OR: 0.98), female (OR: 1.44), junior high school (OR: 1.28), senior high/technical school (OR: 1.37), college or above (OR: 1.71), willingness to live with children (OR: 0.85), family harmony (OR: 0.53), filial piety perceived (OR: 0.81), raising children to ensure eldercare (OR: 0.85), children unreliable for LTC (OR: 1.33), older adults are a family burden (OR: 1.11), feeling of loneliness (OR: 1.11), knowledge of eldercare institutions (OR: 1.87), opinions about eldercare institutions (OR: 2.82), amount of monthly pension (OR: 1.16), amount of savings for old age (OR: 1.13), self-assessed economic status (OR: 0.82); 2) Rural: female (OR: 0.80), married (OR: 1.21), family harmony (OR: 0.71), filial piety perceived (OR: 0.82), raising children to ensure eldercare (OR: 0.74), children unreliable for LTC (OR: 1.32), older adults are a family burden (OR: 0.91), knowledge of eldercare institutions (OR: 1.52), opinions about eldercare institutions (OR: 1.68), IADL score (OR: 0.95), eldercare institutions in the community (OR: 0.64), self-assessed economic status (OR: 0.82)
(18) Block 1	Spain	729 older adults > 55 years old from a Spanish population sample	To examine ex-ante and current preferences for housing (in old age) and its suitability, given current and future needs and characteristics	**Single question (open ended)** DV “If in the future you were to suffer a restriction in some activities of daily living such as walking, bathing, taking medication or using the telephone, where would you prefer to live?” IV: gender, age, housing price, size, income, saving, education, health and disability	Preferred type of residence in case of old-age dependency: 1) In my own home (men: 78.71%, women: 77.78%), 2) In a nursing home or similar (men: 17.67%, women: 15.6%), 3) In a relative’s home (men: 3.61%, women: 6.62%) Willingness to move: housing suitability changes with age, women were less likely to prefer moving, 10% increase in housing asset reduced probability of moving by 1.5% Housing improvements: 51% of the 55–60 age-group were prepared to make structural improvements to their dwelling compared to only 18% in the over-80 s group
(68) Block 1	China	1186 people over the age of 40	To describe willingness of urban and rural residents to enter care homes and to examine personal factors which impact willingness	**Binary choice (yes/no)** DV: “Would you be willing to reside in a care home in the future?” IV: socio-demographics (age, gender, education, marital status, monthly income, occupation), physical health (chronic diseases), lifestyle and behavior (current residence, smoking, drinking, physical activity)	34.8% were willing to enter a care home (41.6% of urban participants and 25.8% of rural participants) Willingness associated with*: Being employed (OR: 1.69), Education – high school (OR: 1.99), education – undergraduate or junior college (OR: 1.94), Higher monthly income (OR: 1.72)
(7) Block 1	USA	1503 adults of the general population between ages 40 to 70	To disentangle the distribution of preferences for LTC along two dimensions: (1) care at home vs. a place other than home, (2) care provided by family vs. care by others	**Rating** DV: Preferences for care provided by 1) your family in your home, 2) your family in their home, 3) paid caregivers in your home or community, 4) a resident of an assisted living facility, 5) a resident of a nursing home IV: self-rated health, educational attainment, age, self-reported race, marital status, household income, respondent’s sex, additional questions to service familiarity	Preferred type of LTC: 1) your family in your home (63.5%), 2) your family in their home (32.8%), 3) paid caregivers in your home or community (46.6%), 4) a resident of an assisted living facility (30.1%), 5) a resident of a nursing home (10.0%) Preference for care at home: Female (OR: 1.39), very good health (0.39) Preference for care by kin: Education post high school (OR: 1.78), female (OR: 1.55), knowledge of services (OR: 0.86)
(34) Block 1	Spain	2535 older adults aged 65 years and over living in private dwellings	To shed light on the preferred residential and care arrangements in later life	**Questions with pre-specified categories** DV: preferred setting in case of not needing any kind of support/care; this setting in case of suffering any disability, which may impede the normal development of daily routines IV: socio-demographics, income, health status, current living environment, psychological and attitudinal factors	Preferred residential solution in old age dependency: 1) In own home (21%), 2) Co-residence—relative’s home (56%), 3) Co-residence – others (2%), 4) Institutionalization (21%) Preference for co-residence*: Widowed (Coeff: 0.09), living at children’s home (Coeff: 0.14), primary school (Coeff: 0.15), savings and assets (Coeff: 0.28), health status (Coeff: 0.11), preference for informal care (Coeff: -0.07), older people need constant support (Coeff: -0.12), older people are active and enjoy life (Coeff: -0.19), older people support other family members (Coeff: -0.14), older people do not have the support of society (Coeff: -0.08) Preference for institutionalization*: Gender (Coeff: -0.06), age 65 (Coeff: 0.06), age 80 (Coeff: −0.05), widowed (Coeff: -0.05), university (Coeff: 0.07), health status (-0.08), receiving formal care (Coeff: 0.09), loneliness (Coeff: 0.06), family satisfaction (Coeff: -0.09), preference for informal care (Coeff: -0.14), preference for paid care (Coeff: 0.07), older people need constant support (Coeff: 0.06), older people are a burden (Coeff: 0.09), older people do not have the support of society (Coeff: -0.08)
(60) Block 1	China	1090 participants from 4 Chinese cities	To explore and make theoretical sense of older people’s LTC needs; to identify the factors influencing LTC needs	**Single question with pre-specified categories **DV: “Which mode of LTC would you like to choose?” – 1) family care, 2) Home- and community- based services (HCBS), 3) institutional care IV: predisposing characteristics (age, gender, educational level, marital status, region), enabling factors (income, number of children, contact frequency with children), need factors (IADL, number of diseases), psychosocial factors (intergenerational relationships, unmet care service needs, self-image)	Preferred LTC option: 1) 75.3% family care, 2) 16.7% HCBS, 3) 8.0% institutional care Family care vs. HCBS*: Region – BJ (OR: 0.474), Region – GZ (OR: 0.265), Region – HB (OR: 0.382), Number of children (OR: 1.268), Unmet care needs (OR: 0.936), Self-image (OR: 1.027) Institutional care vs. HCBS*: Currently not married (OR: 2.362), Region – GZ (OR: 0.138), Intergenerational relationships (OR: 0.676), Unmet care service needs (OR: 0.912), Self-image (OR: 1.044)
(50) Block 1	Slovenia	930 people ≥ 50 years old	To test the willingness of the elderly to accept various housing options and the attitudes toward the different options	**Rating** DV: “Have you considered moving in the future?” 1) Old people’s home, 2) Sheltered housing, 3) Senior co-housing, 4) Living in a multigenerational residential building, 5) Household groups, 6) Living with another family or individual, 7) Living with a caregiving family for older people IV: education, revenue, current health status, residence, age, type of settlement	Living arrangement (% acceptable): 1) Old people’s home (71.7%), 2) Sheltered housing (55.6%), 3) Senior co-housing (25.8%), 4) Living in a multigenerational residential building (32.0%), 5) Household groups (30.6%), 6) Living with another family or individual (11.3%), 7) Living with a caregiving family for older people (7.5%) Associated with*: Education, revenue, current health status, age
(54) Block 1	Germany	1006 older adults aged 65 and over	To investigate the factors associated with preferences for LTC settings in old age in Germany	**Rating** DV: “When care is needed, I would like to be cared for…” 1) at own home, 2) in relatives’ homes, 3) in assisted living, 4) in nursing home/old age home, 5) in a foreign country IV: age, sex, living situation, region, education, place of birth, having children, status of health insurance, providing informal care to family or friends	Preferred LTC settings: 1) Home care (n = 866, 87.2%), 2) Care in relatives’ homes (n = 319, 32.3%), 3) Care in assisted living (n = 534, 55.6%), 4) Care in nursing home/old age home (n = 310, 32.0%), 5) Care in a foreign country (n = 54, 5.5%) Preference for home care*: provided care for family/friends (OR: 1.600), level of care (OR: 0.189), self-rated health (OR: 0.762) Preference for care in relatives’ homes*: female (OR: 0.506), living situation (OR: 0.559), children (OR: 1.610), status of health insurance (OR: 1.566), provided care for family/friends (OR: 1.468), self-rated health (OR: 1.192) Preference for care in assisted living*: apprenticeship/full-time vocational school (OR: 2.984), professional school or trade and technical school (OR: 2.666), university/Fachhochschule/school of engineering (OR: 3.494), level of care (OR: 1.900) Preference for care in nursing home*: German-born (OR: 1.782), self-rated health (OR: 0.850) Preference for care in foreign country*: age (OR: 0.902), apprenticeship/full-time vocational school (OR: 0.246), German-born (OR: 0.184)
(59) Block 1	China	670 adults aged 60 and above in the Hezuo community in Chengdu, China	To describe Chinese older adults’ willingness to enter LTC facilities; to examine individual factors associated with the willingness	**Binary choice (yes/no) **DV: “Are you willing to enter into one of these LTC facilities that integrate medical and social services in the future?” IV: socio-demographics (age, gender, marital status, education, occupation, income, insurance, living condition, being sick in last two weeks, number and type of chronic diseases, hospitalization in prior year), general well-being index, social support	11.9% were willing to enter LTC facilities for meeting their medical and social service needs. Among those who were willing to enter LTC facilities, 81.2% would prefer going to a facility within 30-min walking distance from their current residence, 82.5% indicated the need of nursing care, and 90.0% expected a partnership between the LTC facility and a large hospital
(13) Block 1	USA	98 respondents with a mean age of 62	To explore attitudes toward and preferences for living in the newly emerging place type of assisted living facilities in comparison to nursing homes	**Rating** DV: “In the future, how favorable or unfavorable would you feel about living in the places indicated below?” 1) assisted living, 2) nursing home, 3) their own homes IV: age, gender, educational status, current living arrangements, occupation, current work status	Preferred LTC option: Living at home was most favorable (mean = 1.06, SD = 0.43), followed by assisted living facilities (mean = 2.43, SD = 1.02) and nursing homes (mean = 4.44, SD = 0.75) Willingness associated with*: Higher household income (OR: 4.55), insurance of urban resident basic medical insurance (OR: 4.80), unemployment (OR: 0.48)
(36) Block 1	USA	499 “boomers” (age 51–71 years) in the state of Washington (Japanese-Americans compared to non-Japanese Americans)	To investigate perceptions about future LTC needs and preferences among the baby boomer generation of Japanese-American (JA) relative to non-JA older adults	**Rating** DV: LTC preferences, knowledge about LTC, LTC experience, expectation for LTC, LTC financial planning IV: age, sex, race, marital status, education and household income, geographical distance to children	Preferred LTC option in old age for JA: remain in their own home (24.03%), move to retirement community (18.78%), move to continuing care retirement community (17.26%), move to a skilled nursing or assisted living (4.57%), live with family member or close friend (4.57%), unsure (38.58%) LTC knowledge: JA were more knowledgeable about the cost of a month’s stay at a NH, more aware that these services would have to be paid out of pocket and more knowledgeable about the prevalence of Alzheimer’s disease than non-JA Financial planning: JA participants were 3.15-fold more likely to have an IRA, 401-K account or an annuity and 2.55-fold more likely to have a LTC insurance policy than non-JA
(14) Block 1	USA	427 Korean-American adults aged 60 years or older	To explore predictors of willingness to use a nursing home in Korean American elders	**Binary choice (yes/no)** DV: Willingness to move to a nursing home IV: Predisposing factors (age, gender, educational attainment, length of residence in the USA), Potential health needs (medical conditions, functional disability, self-perceived health), Network-related enabling factors (marital status, living arrangement, family network, relative living in a NH)	44.7% of the sample were willing to move to a nursing home in the future. Determinants of the willingness to use a nursing home*: poorer self-perceived health (OR: 1.46), having someone close living in a nursing home (OR: 2.80)
(16) Block 1	Korea	1168 older Korean adults aged 65 or older and their primary caregivers	To examine predictors of older adults’ and primary caregivers’ willingness to use LTC services	**Binary choice (yes/no)** DV: “Who do you think should take care of older parents if they become frail and need care?” 1) adult children only 2) both adult children and the government 3) the government only; Willingness to use LTC services—1) home care services 2) nursing home care services IV: predisposing factors (age, sex, marital status, education, employment, relationship to older adults, responsibility for care), enabling factors (number of children, monthly family income, geographic region), need factors (self-rated health, impairments in ADLs and IADLs, cognitive function, chronic conditions, care burden)	Willingness to use (older adults): home care (32.4%), nursing home care (16.6%) Willingness to use (primary caregivers): home care (40.5%), nursing home care (32.5%) Home care preference associated with*: (1) Older adults: elders saying care responsibility has children and government (ß = 0.76), caregivers saying care responsibility lies with children and government (ß = 0.55), (2) Primary caregivers: married (ß = 0.79), self-rated health as bad (ß = 0.58), care burden (ß = 0.02), caregivers saying care responsibility lies with children and government (ß = 1.53) Nursing home care preference associated with*: (1) Older adults: female (ß = −1.05), Number of children 5 + (ß = -1.30), monthly family income less than 1100 won (ß = 0.77), MMSE score 24–30 (ß = 1.01), Elders saying care responsibility lies with children and government (ß = 1.53), (2) Primary caregivers: middle school or more (ß = 1.06), Number of children 5 + (ß = −0.77), Elders saying care responsibility lies with children and government (ß = 0.62), care burden (0.03), primary caregivers saying care responsibility lies with children and government (ß = 0.96)
(8) Block 1	Korea	1850 people aged ≥ 65 years with > 1 limitation in ADLs or IADLs or scores < 20 on the MMSE	To explore the factors influencing the intention of Korean elderly to use LTC facilities	**Binary choice (yes/no)** DV: Intention to use LTC facilities IV: Predisposing factors (age, sex, marital status, education level, religion), Enabling factors (number of children, family structure, family income, place of residence), Need factors (self-rated health, ADLs, instrumental ADLs, cognitive level, comorbidity)	18.8% (347) respondents expressed the intention to use LTC facilities. Stronger intention associated with*: younger age (OR: 1), Cristian religion (OR: 1.44), fewer children (OR: 1), lower family income (OR: 1), more chronic comorbidity (OR: 1.59)
(2) Block 1	USA	169 well-educated, relatively affluent older caregivers	To examine gender differences in attitudes about home and community-based services, service use, interest in services, and barriers to service use	**Rating** DV: Interest in specific services, Attitudinal questions: preference for informal care, belief in caregiver independence, acceptance of government services IV: age, sex, race, marital status, education, income, self-rated health, information about the care-recipient (age, sex, relationship to caregiver, ADLs, IADLs, frequency of help)	Service interest: Information about community and in-home support services (77.2%), Information about potential financial problems (78.9%), Support from other caregivers (61.1%), Respite care for family member (59.3%), Hospice Care (60.0%), Individual counselling (43.4%), Information about stress management (58.7%), Information about legal options (69.1%) Preferences for Informal care: I would rather use community services than ask for help from family (38.7%), I would rather ask family for help than use community services (56.7%) Belief in Caregiver Independence: I am proud of being able to care for my relative with little help from community services (63.1%), I think I should be able to care for my relative without help from community services (29.3%) Acceptance of Government Services: It is the government’s responsibility to help me find ways to care for my relative (21.1%), I believe the government should support more community services to help families care for persons at home (78.1%)
(46) Block 1	China	517 elderly from three rural villages (60 years and above)	To explore the factors influencing the willingness of the elderly to receive institutional and community-based eldercare in rural communities in China	**Single question with pre-specified categories** DV: “Which are you willing to choose?” Options: (0) home-based care, (1) living in a nursing home, (2) living in a senior care unit of a hospital, (3) community-based care) IV: socio-demographic information, physical disease, depression, anxiety, daily living activities, concerns toward eldercare in future	Preferred LTC options: 78.3% are willing to receive home-based eldercare, 10.8% institutional eldercare and 8.5% community-based eldercare. Major concerns toward 0: lack of care ability and loss of contact with family members Major concerns toward 1: unaffordable services and fear of being abandoned by children Major concerns toward 3: unaffordability and lack of necessary care
(4) Block 1	USA, Germany	558 American seniors and 772 German community-dwelling older adults	To investigate preferences for future support by older adults who live an independent life in the community	**Questions with pre-specified categories** DV: Preferences for short- and long-term care: 1) getting help from relatives 2) getting help from friends and acquaintances 3) moving in with relatives, 4) getting home health care 5) moving to an assisted living facility 6) moving to a nursing home IV: Deficits in everyday competence, socioeconomic status, informal network, previous receipt of personal care, age, gender, marital status, perceived availability of options	Short-term care: 1. Exclusive use of informal support: USA (19.0%), GER (32.4), 2. Exclusive use of formal support: USA (17.5%), GER (13.7%), 3. Use of mixed support: USA (59.5%), GER (51.7%), 4. No support preferences: USA (4.1%), GER (2.2%) Long-term care: 1. Exclusive use of informal support: USA (15.2%), GER (24.9%), 2. Exclusive use of formal support: USA (36.0%), GER (29.2%), 3. Use of mixed support: USA (43.2%), GER (39.6%), 4. No support preferences: USA (5.6%), GER (6.3%)
(51) Block 3	Poland	214 senior citizens ≥ 55 years of age	To identify the willingness of pensioners to move from apartment/house to one located in a housing estate	**Binary choice (yes/no)** DV: willingness to change housing adapted to the needs resulting from mobility limitations, preferences for facilities at senior housing estate IV: gender, age, place of residence, family situation	Willingness to change place of residence: village (yes: 15.5%), city up to 50,000 residents (yes: 12.6%), city up to 100,000 residents (yes: 20.4%), city up to 250,000 residents (yes: 4.9%), city over 250,000 residents (yes: 46.6%) Most popular facilities were: 24 h on-call medical car–button in the apartment (63%) and 24 h medical care–on call duty at estate (60%)
(57) Block 1	China	505 rural single seniors from Shandong, China	To examine the gender difference toward utilization willingness of institutional care among rural single seniors	**Binary choice (yes/no)** DV: “Which way of elder care are you willing for?” – institutional care coded as yes, rest coded as no IV: age, gender, education, number of children, relationship with children, residence, living arrangements, household income, family size, physical health status (self-rated health, non-communicable chronic diseases, ADL), psychological well-being (social support, psychological stress)	5.7% of rural single seniors were willing to use institutional care (9.3% of single male and 3.5% of single female seniors) Associated with*: female (OR: 0.19), living arrangement–with children or others (OR: 0.13), psychological stress (OR: 0.005)
(56) Block 1	China	3923 seniors in Shandong	To compare the willingness to use institutional care between empty-nest and non-empty-nest seniors in China	**Binary choice (yes/no)** DV: “Which endowment way are you willing for?”—Seniors’ willingness for institutional care (No = home-based care, community endowments or others) IV: gender, age, education, past occupation, marital status, number of children, relationship with children, residence, self-reported health status, psychological stress, ADL, non-communicable diseases, household income	8.5% of seniors indicate willingness for institutional care. Associated with*: Non-empty nester (OR: 1.0), Empty-nest single (OR: 5.301), Empty-nest couple (OR: 1.547), Junior school or above (OR: 1.617), Poor relationship with children (OR: 2.504), Rural Residence (OR: 0.546), ADL III (OR: 0.577), Household income Q3 (OR: 1.612), Household income Q4 (OR: 2.065)
(49) Block 3	USA	Data set with 244,718 residents ≥ 65 years	To group residents according to similarities in preferences; to determine the factors that predict membership in these groups	**Rating** DV: nursing home preferences (The MDS preference assessment was used, which assesses the importance of 16 daily care and activity preferences using a 5-point Likert scale.) IV: race, gender, cognition, ethnicity, depression	4 classes were determined: 1. “important” (38.3%), 2. “activity” (27.1%), 3. “care” (24.1%), 4. Unimportant (10.4%) Activity vs. care*: African American (OR: 1.6), Hispanic (OR: 2.1), Other (OR: 1.5), female (OR: 1.7), depressed (OR: 0.7), moderate cognitive impairment (OR: 1.5), severe cognitive impairment (OR: 3.1) Important vs. care*: African American (OR: 2.35), Hispanic (OR: 2.95), Other (OR: 1.5), female (OR: 1.6), depressed (OR: 0.65), severe cognitive impairment (OR: 2.0) Important vs. unimportant*: African American (OR: 2.15), Hispanic (OR: 2.1), female (OR: 2.05), depressed (OR: 0.5), severe cognitive impairment (OR: 0.6) Unimportant vs. care*: Other (OR: 1.8), moderate cognitive impairment (OR: 1.6), severe cognitive impairment (OR: 3.4) Activity vs. unimportant*: Hispanic (OR: 1.55), female (OR: 2.1), depressed (OR: 0.5) Activity vs. important*: African-American (OR: 0.7), severe cognitive impairment (OR: 1.5)
(47) Block 1	China	306 parents aged > 49 years who lost their only child in these communities: Jinghu, Jiujiang, Yijiang and Sanshan	To understand the elderly care needs of parents who lost their only child	**Questions with pre-specified categories** DV: “Which mode of elderly care would you like to choose in the future?” – 1) community or home care, 2) institutional, community or home care) and “Whom do you want to look after you in the future?” – 1) family, 2) nurse/nursing home, 3) volunteer) IV: sex, educational level, marital status, having grandchildren, number of chronic diseases, self-rated health status, monthly income, social support rating scale (SSRS), European Quality of Life-5 Dimensions (EQ-5D), Geriatric Depression scale (GDS)	Preferred LTC options: 1) 47.4% parents chose community or home care, 2) 52.6% chose institutional care. Preference associated with*: Higher GDS score (OR 1.179)
(28) Block 1	Germany	1445 respondents ≥ 45 years from the general population	To assess wishes and expectations regarding family care	**Questions with pre-specified categories** DV: Willingness to make use of family resources in case of a care dependency IV: sex, age, family status, education level, family income, living situation, care experience	In case of own need: 62.9% of participants would prefer care provided by relatives and 56.7% would prefer professional care Willingness to use family care associated with*: living together with a partner (OR: 3.65), having children (OR: 3.12), having personal care experiences (OR: 1.35), image of being a burden at old age (OR: 0.79) Perceived willingness of relatives to provide care was influenced by*: geographical distance > 50 km apart (OR: 0.30), spoken with child about wishes for old age (OR: 2.11), spoken with child about how care should be financed (OR: 1.90), being female (OR: 0.59), spoken with partner about wishes for old age (OR: 2.31)
(20) Block 1	China	186 young, 161 middle-aged and 185 older Chinese in Hong-Kong	To explore age-cohort differences on attitudes and intention toward old age home placement	**Rating** DV: Intention to enter/refer to old age home, attitudes toward old age homes and older people IV: age, gender, marital status, educational attainment	Intention of referring to old age home: young adults (mean: 3.15), middle-aged (mean: 3.31) Intention of entering old age home: older Chinese (mean: 2.08) Intention of referring/entering old age home*: 1) Young adults: beliefs about independence (ß = 0.21), attitudes toward old people (ß = 0.18), attitudes toward old age home (ß = 0.42); 2) Middle-aged: attitudes toward old age home (ß = 0.30); 3) Old-aged Chinese: attitudes toward old age home (ß = 0.33)
(12) Block 1	Taiwan	593 elderly people aged ≥ 65 years and 587 caregivers	To examine the preferences of the elderly and their primary caregivers in LTC arrangements	**Single question with pre-specified categories** DV: “If you (your elderly family member) needed LTC services, taking every factor into practical consideration, which kind of LTC would you feel more inclined to accept?” 1) Home care 2) Community care 3) Institutional care IV: gender, age, educational level, marriage status, ethnic origin, religion, residency, monthly income, perceived health status, care resources, receipt of a monthly stipend from the government, care experience	Preferred LTC option (elderly): 1) Home care (73.7%) 2) Community care (9.6%) 3) Institutional care (16.7%) Preferred LTC option (primary caregiver): 1) Home care (68.1%) 2) Community care (12.2%) 3) Institutional care (19.7%) Factors influencing preference for institutional care*: 1) Elderly: ethnic origin—China (OR: 2.15), residential location – other areas (OR: 1.81), No government subsidy (OR: 1.78); 2) Primary caregiver: ethnic origin – China (OR: 2.24), elementary education (OR: 3.79), not harmonious relationship to elderly (OR: 1.66) Factors influencing preference for community-based care*: 1) Elderly: age 65–74 years (OR: 3.12); 2) Primary caregivers: ethnic origin – China (OR: 2.26), not harmonious relationship to elderly (OR: 1.84), no care difficulties (OR: 1.90)
(67) Block 1	China	3260 elderly people aged ≥ 60 years	To provide a reference to enable policymakers to optimize resource arrangement and ensure the sustainability of China’s old-age security policy	**Questions with pre-specified categories** DV: Combination of medical care and Pension in LTC facilities (CMCP)—1) “Do you want to live in an LTC facility with CMCP?”, 2) “If you live in a CMCP facility, what kind of service would you like to receive?” IV: predisposing variables (age, gender, residence, current living arrangement, educational level), enabling factors (occupation, marital status, economic status, primary caregivers, number of children, medical insurance), personal needs variables (activities of daily living, feelings of loneliness, self-rated health status)	Preferred LTC options: 12.89% wanted to be admitted to a CMCP nursing facility, 82.01% preferred home-based care and 5.1% preferred community care. Significantly associated with*: Residence – Town (OR: 1.74), Residence – Rural (OR: 2.16), Not married (OR: 1.64), Economic status/expenditures are balanced (OR: 1.64), Economic status – income exceeded (OR: 2.28), Primary caregiver: spouse (OR: 0.32), Primary caregiver: others (OR: 1.77), ADL: relatively independent (OR: 2.13), ADL: Disability (OR: 3.69)
(62)Block 3	China	82 senior citizens living in their original communities	To identify senior citizens’ requirements related to ‘embedded retirement facilities’ (ERFs) – small-scale, multi-functional and community-based care facilities	**Ranking** Ranking of the four main aspects of services (in cursive), followed by ranking of 22 detailed services related to each main aspect	1. Health care (1. Regular health examination, 2. Health counselling, 3. Going to the hospital with someone accompanied, 4. General practitioner, 5. Psychological counselling); 2. Rehabilitation and entertainment (1. Fitness exercises, 2. Chess and cards playing, 3. Singing and dancing, 4. Massage, 5. Post-operative nursing); 3. Daily life assistance (1. Canteens for senior citizens, 2. Room cleaning, 3. Meals on wheels, 4. Laundry, 5. Day care, 6. Purchasing, 7. Full-time nursery, 8. Legal assistance); 4. Culture-related activities (1. Reading, 2. Seminars, 3. Painting and calligraphy, 4. Handmade activities)
(48) Block 1	China	1308 elderly from Chengdu, Chongqing, Guizhou, and Hubei province	To explore the factors that affect LTC needs of the elderly	**Single question with pre-specified categories** DV: “Which LTC way do you want to choose?”–1) home care, 2) community-based care, 3) institutional care IV: predisposing characteristics (age, gender, education level, marital status, regions), enabling factors (income level, quantity of children, frequency of connection with children), need factors (IADL, quantity of chronic diseases) and psychological factors (intergenerational ties, unmet needs for LTC, self-image evaluation)	Preferred LTC option: 75.3% chose home care, 16.6% community-based care and 8.0% institutional care. Institutional care vs. community-based care*: unmarried (OR 2.4801), region–GZ (OR: 0.1449), intergenerational relationships (OR: 0.7098), unmet care service needs (OR: 0.9576), self-image evaluation (OR: 1.0962) Home care vs. community based care*: Region–CQ (OR: 0.4977), Region–GZ (OR: 1.2782), Region–HB (OR: 0.4011), number of children (OR: 1.3314), unmet care service needs (OR: 0.9828), self-image evaluation (OR: 1.0783)
(58) Block 1	China	14,373 participants aged 60 and above	To assess the effects of health status and living arrangements on LTC models among the elderly of Xiamen, China	**Single question with pre-specified categories** DV: “Where do you most want to receive LTC?”–1) family care, 2) community care, 3) institutional care) IV: predisposing factors (age, gender, occupation, education), enabling variables (living arrangements, residence location, marital status, medical insurance, family income, number of children), need variables (ADL, chronic diseases, self-rated health, life satisfaction, feelings of aloneness)	Preferred LTC options: 86.37% home care, 2.86% institutional care, 10.77% community-based care Associated with*: Health status–relatively independent (OR: 1.64), health status–disability (OR: 3.95), living arrangement with child (OR: 0.54), living arrangement–spouse and child (OR: 0.67), living arrangement–others (OR: 0.60),

^1^This column includes the content-related classification of each study into one of the four main entities (“blocks”)

Note: *only statistically significant results are presented* (*), DV = dependent variable, IV = independent variable

The included studies (*n* = 66) investigated different facets of LTC. For this systematic overview, studies were grouped into four main entities:

1. Preferences for different LTC options and factors influencing these preferences: Twenty-eight studies exclusively focused on the preferred type of care in case the participants became care-dependent and its influencing factors (e.g., age, gender) that might explain respondent’s preferences. Participants were asked to choose their preferred LTC option from a set of pre-specified categories or show their level of agreement to a set of statements concerning different LTC options (usually Likert scales). Binary-choice questions investigated respondent’s willingness to make use of only one type of LTC, e.g., nursing home care.
2. Suitability of different types of care services and settings for hypothetical patient outcomes and factors influencing such preferences: In the majority of the studies (*n* = 11), hypothetical vignettes were used that depicted individuals in need of care, while the type and severity of impairment differed between studies. Physical and cognitive impairments were mostly compared. Participants were then asked to put themselves in the position of the hypothetical person in need of care and state the appropriate LTC option. The influencing factors in most studies were then analyzed.
3. Preferences for the design and structure of specific LTC services: Twenty-one studies focused on a singular type of LTC to make preference-based suggestions for the improvement of specific LTC service designs. Participants were mostly asked to make trade-offs between different attributes of the depicted LTC service, e.g., the cost and care time per day of two home-based care packages. DCE and conjoint analyses were most frequently used. When reported, the number of included attributes in the experimental designs ranged from four to ten. Studies either focused on informal care, home-based, and community-based care or LTC facilities.
4. Impact of LTC services to value quality of life or quality of care instruments: Preference-based instruments typically incorporate a scoring algorithm that has been elicited by using various SP methods and can be used to measure caregiver’s outcomes.

### Block 1.1: Preferences for different LTC options

Twenty-eight studies exclusively asked respondents for their preferred LTC option in case of a care dependency situation and investigated the impact of a variety of independent variables on the choices of respondents. The majority of these studies were conducted in China (*n* = 12) and the USA (*n* = 7). The most preferred care option by almost all (60 to 85%) participants was home care, either provided by relatives or professionals (Chung et al. 2008; Costa-Font et al. 2009; Eckert et al. 2004; Fernandez-Carro 2016; Fu et al. 2017; Filipovič Hrast et al. 2019; Hajek et al. 2017; Imamoğlu and Imamoğlu 2006; Laditka et al. 2001; Liu et al. 2019; Rong et al. 2019; Spangenberg et al. 2012; Wei and Zhang 2020; Zeng et al. 2019; Zhang et al. 2017; Fisher 2003; Kim and Choi 2008; Wang et al. 2004; Iwasaki et al. 2016). Some studies further differentiated between receiving care and living in one’s own home or the relative’s home, of which the option of remaining in one’s own home was preferred by all except in the study by Fernández-Carro (2016). Here living in the relative’s home was preferred by 56% of respondents compared to 21% of respondents preferring to be cared for in their own home. Pinquart and Sorenson (2002) found that older respondents preferred informal or mixed support for short-term care dependency and more formal assistance in the case of LTC needs. Seven studies investigated preferences for community-based care. On average, 5 to 10% of the respondents preferred to use community services in case of a care dependency (Chung et al. 2008; Liu et al. 2019; Wei and Zhang 2020; Zhang et al. 2017). In the remaining three studies, willingness to use community services was higher, documenting up to 38.7% (Laditka et al. 2001; Rong et al. 2019; Zeng et al. 2019).

The third LTC option most often investigated was institutional care, particularly, respondent’s intention or willingness to enter a nursing home when in need of care. Acceptability of institutional care varied greatly between studies. In most studies, 2 to 20% of respondents expressed their willingness to enter a care home in the future (Chung et al. 2008; Costa-Font et al. 2009; Eckert et al. 2004; Fernandez-Carro 2016; Fu et al. 2019; Liu et al. 2019; Wei and Zhang 2020; Zeng et al. 2019; Huang et al. 2018; Kim and Kim 2004; Qian et al. 2017, 2018; Chou 2010; Wang et al. 2004; Iwasaki et al. 2016). The lowest acceptability rate was found in a Chinese survey with 14,373 participants aged 60 and above with a willingness of less than 3% to enter a nursing home (Zhang et al. 2017). A study by Hrast et al. (2019) (72%) in Slovenia recorded the highest acceptability rates.

### Block 1.2: Factors influencing preferences for different LTC options

To better understand the LTC choices of respondents, many of the included studies investigated the influence of a variety of independent variables on such choices. Which independent variables were looked at was, however, heterogenous across studies and either chosen research-focused (*n* = 19) or with the help of Anderson’s model of health services utilization (*n* = 9). When studies applied Anderson’s model, independent variables were most often grouped into *predisposing* (e.g., age, sex, nationality, educational level), *enabling* (e.g., income, number of children, family network, social support) and *need* (e.g., self-rated health, cognitive function, number of chronic diseases) factors on the choices of respondents. A list of independent variables that each study investigated can be found in Table 2.

Preferences for home care were positively associated with lower self-rated care (OR: 1.3), no need of care (OR: 5.5), and providing care for family members or friends (OR: 1.6) in a German study by Hajek et al. (2017). Expectations of reciprocity and strong family bonds might explain such preferences. In the Chinese study by Liu et al. (2019), older adults living in rural areas preferred home-based services and receiving support from family members. Eckert et al. (2004) found a higher education (OR: 1.78) and being female (OR: 1.55) increased preference for care by kin. Contrastingly, Fernandez-Carro (2016) found that factors like being widowed (Coeff: 0.09), a low educational (Coeff: 0.15), and financial profile (Coeff: 0.28), and already living at children’s home (Coeff: 0.14) significantly increased the likelihood of choosing co-residence at a relative’s home. Compared to home care, those respondents who preferred community-based care were in need of visiting a medical care team (OR: 2.12) and needed self-care information (OR: 4.39) (Chung et al. 2008). A study involving 169 older caregivers in the USA found a significant gender difference for being able to afford to pay for services. It found that almost 60% of male respondents and 33.9% of female respondents would rather use community services than ask family for help (Laditka et al. 2001).

When it comes to respondents’ willingness to use institutional care services, several studies found that poorer health status was positively associated with such willingness (Wei and Zhang 2020; Rong et al. 2019; Zhang et al. 2017; Jang et al. 2008; Kim and Kim 2004; Hajek et al. 2017). Marital status (unmarried or widowed) additionally positively influenced respondents’ willingness to enter a care home (Wei and Zhang 2020; Zeng et al. 2019; Rong et al. 2019; Qian et al. 2018; Fernandez-Carro 2016). Income was found to have a statistically significant impact on respondents’ choice to receive institutional care in several studies. Studies by Wei and Zhang (2020), Qian et al. (2018), and Dong et al. (2020) found that respondents with a higher income were more likely to prefer an institutional setting. However, Kim and Kim (2004) and Kim and Choi (2008) found that the opposite was true. A higher education was also found to positively influence respondents’ willingness to enter a care home (Wei and Zhang 2020; Qian et al. 2018; Fernandez-Carro 2016; Chou 2010; Dong et al. 2020). Fewer children or a poor relationship with one’s children increased respondents’ willingness to move to a LTC facility in the future (Kim and Kim 2004; Qian et al. 2018). Having an acquaintance already living in a nursing home (OR: 2.80) additionally increased respondents’ willingness to enter a nursing home (Jang et al. 2008). A Chinese study investigating age-cohort differences on the intention toward old age home placement found that middle-aged and older Chinese respondents tended to have more positive attitudes toward old age home and were willing to enter a nursing home than younger participants (Tang et al. 2009).

**Table 3:** Included studies using contingent valuation techniques (*n* = 6)

Study^1^	Country	Population	Study objective	Type of measurement	Main results
(45) Block 3	China	536 older adults with different service needs living in Hong Kong	To explore the policy implications of introducing co-payments for LTC services in Hong Kong	WTP: Payment scale format in questionnaire surveys for elderly respondents. The voucher was put as an alternative mode of service provision to the current provision-in-kind Other variables: Attitude toward the voucher scheme, financial condition, family support, sociodemographic characteristics, self-care ability	WTP in HK $ (%): 0 (4.1%), 1–100 (16.6%), 101–300 (9.0%), 301–500 (7.1%), 501–700 (7.6%), 701–900 (10.1%), 901–1100 (9.7%), 1101–1500 (12.1%), 1501–1700 (5.4%), 1701–2250 (10.3%), 2251 or more (8.0%) Contributing factors to a higher WTP: positive perception of voucher schemes, higher income, family support, old age
(26) Block 3	France	201 informal caregivers providing care to elderly care recipients with cognitive impairments	To explore the influence of intangible impacts of caring on both informal caregivers' ability to estimate their WTP to be replaced and their WTP value	Derived payment card framework (Intangible impacts of caring is approximated by informal caregiver’s WTP to be replaced for 1 h of care)	WTP in € (% of respondents): ‘I can’t estimate it’ (45%), ≤ €13 (19%), between > €13 and ≤ €18 (23%), > €18 (13%)
(22) Block 3	UK, Spain, Sweden, USA	517 primary carers of patients with Alzheimer’s disease	To explore the value of informal care; to estimate the WTP of informal carers providing care to a person with Alzheimer’s disease, for reductions in patient care need	Hypothetical scenarios, in which caregivers would be given an opportunity to pay for a reduction in caregiving time	Mean WTP for a 1 h reduction in care need per day was estimated to be £105, £121, £59 and £144 per month for UK, Spain, Sweden and US, respectively
(1) Block 3	Switzerland	109 pairs of patients with dementia and their spouse or relative caregivers	To estimate the WTP of informal caregivers for a reduction of their burden. Payment card format used, followed by an open question of the max. % of wealth participants were willing to pay	WTP for three hypothetical dementia treatments: (1) Prevent future worsening (2) Hypothetical cure (3) Reduction of burden	WTP in monetary amounts and % of wealth: (1) Stabilization (preventing future worsening): Median: 35,000 CHF and 10% of wealth, Mean: 98,119 CHF and 22.5% of wealth; (2) Hypothetical cure: Median: 35,000 CHF and 20% of wealth, Mean: 161,682 CHF and 29.4% of wealth; (3) No burden: Median: 15,000 CHF and 10% of wealth, Mean: 57,500 CHF and 17.0% of wealth
(63) Block 3	China	371 caregivers of dependent elderly people in Shanghai	To evaluate the economic value of informal caregiving in Shanghai; to identify the associated factors of WTP and WTA of caregivers	Max. amount of money willing to pay for 1 h of reduction in caregiving and minimum sum of money willing to accept for 1 extra hour of the least-preferred care task per week	Average maximum WTP for 1 h decreasing in least-preferred care task was 25.31 CNY (SD = 18.10) and minimum WTA for 1 h increasing in least-preferred care task was 38.66 CNY (SD = 22.95)
(27) Block 3	USA	409 clients of HCBS programs in Florida, including 109 proxy respondents	To assess the maximum dollar amount HCBS enrolees or eligible applicants are willing to pay as a measure of the value of the services to them	Sequence of dichotomous choice or close-ended question formats followed by an open-ended follow-up question to elicit WTP. Bidding prices were randomly selected for each respondent from a range of values between $100 and $1200 with $50 increments	Overall WTP for HCBS per month based on types of affordability: 1) Actual affordability: median WTP ($900.55), mean WTP ($933.32); 2) Hypothetical affordability: median WTP ($637.98), mean WTP ($707.13); 3) Self-reported WTP: mean WTP ($564.80)

Note: ^1^This column includes the content-related classification of each study into one of the four main entities (“blocks”)

### Block 2.1: Suitability of different types of care services and settings for hypothetical patient outcomes

Nieboer et al. (2010) elicited the preferences of the general Dutch elderly population (*n* = 1,082) for LTC services by means of a DCE. In each choice set, respondents had to choose the most suitable care package (A vs. B) for a specific patient. Four patient profiles were presented: physically frail elderly, elderly with dementia, and then both groups either living alone or with a partner. Irrespective of the group, the greatest value was attached to a regular care provider and the availability of transportation services. For physically frail elderly, transportation services were deemed most important (*living alone* coeff: -0.572, WTP: €120; *living with a partner* coeff: -0.459, WTP: €76). For older patients with dementia who were *living alone*, the most important attribute was living in an apartment building in close proximity of the caregiver (Coeff: 0.498, WTP: €177). For patients with dementia who were *living with a partner*, the most important attribute was a regular care provider (Coeff: -0,493, WTP: €88). Generally, all services were deemed more important to care-dependent people living alone compared to those living with a partner. Living in a care or nursing home was the least preferred living situation, except for dementia patients living alone.

Additionally, 14 studies examined the suitability of LTC settings for different hypothetical patient outcomes. Of these studies, two applied the conjoint analysis method (Fahey et al. 2017; Robinson et al. 2015), one the time-trade off method (Guo et al. 2015), and the remaining 11 a vignette survey. Fahey et al. (2017) found that respondents placed the greatest weight on reducing strain on family members and wanting to stay at home with support for as long as possible. Robinson et al. (2015) found that the most important attribute of the respondents was going home with care support. Half of the respondents were additionally willing to sacrifice one year or more of their life to be able to stay at home with support. Guo et al. (2015) found that home care was strongly preferred compared to nursing home for mild to moderate physical impairments. Such preferences were found to decrease by 0.04 quality of life weight for every additional ADL impairment. With increasing severity of impairment, especially of a cognitive nature, preferences tended to shift toward nursing home care.

In the remaining studies, vignettes depicting situations with hypothetical older adults in need of care were used. In most cases, the severity of the depicted care dependency was altered in the vignettes and respondents were asked to choose an appropriate LTC option for that person. The majority of the vignette surveys were conducted in the USA and differentiated the hypothetical care dependencies into physical and cognitive impairments (Bradley et al. 2004; McCormick et al. 2002; Min 2005; Min and Barrio 2009; Wolff et al. 2008). In case of IADL needs or a hypothetical hip fracture, respondents preferred to make use of informal or formal home-based care services (Wolff et al. 2008; McCormick et al. 2002; Min 2005; Min and Barrio 2009; Kasper et al. 2000, 2019). While McCormick et al. (2002) found similar preferences between Japanese-Americans and Caucasian-Americans in case of a hip fracture, Min *and* Barrio (2009) found that 83.3% of non-Latino white older adults expressed a preference to rely on formal or paid help compared to 54.6% of Mexican-Americans. In terms of cultural values, 73.3% of Mexican–American respondents agreed that care should be provided by family members and not by outsiders as compared to 32.6% of non-Latino White respondents (Min and Barrio 2009). In a US study by Bradley et al. (2004), in the hypothetical case of cognitive and physical impairment, African-American respondents were more likely to use informal LTC (72.3%). In the hypothetical case of dementia or stroke, preferences shifted toward nursing home care or an exclusive use of formal care services (Min 2005; Wolff et al. 2008; Werner and Segel-Karpas 2016; Kasper et al. 2000). With increasing severity of impairment, preferences tended to also shift toward nursing home care (Carvalho et al. 2020; Santos-Eggimann and Meylan 2017). Adult children were more likely to recommend moving to a retirement facility than older adults (Caro et al. 2012).

### Block 2.2: Factors influencing preferences for LTC settings for hypothetical patient outcomes

In case of a hip fracture, age (OR: 1.07) and being female (OR: 2.24) increased the likelihood of Japanese-American respondents choosing home healthcare (McCormick et al. 2002). In a sample of 144 Korean-Americans, being female (OR: 0.09), having a higher education (OR: 0.76), and having stronger traditional values (OR: 0.79) significantly increased the likelihood of choosing an informal instead of formal care arrangement for a possible hip fracture (Min 2005). With increasing severity of incontinence, the proportion of people choosing institutional care instead of home-based care increased significantly. Personal incontinence of respondents had no significant impact on their choice of LTC option (Carvalho et al. 2020). In three studies, race played a significant role in explaining preferences. Being African-American (OR: 2.41) and Mexican–American (OR: 4.6) increased the likelihood of turning to an informal caregiver in case of a care dependency situation (Bradley et al. 2004; Min and Barrio 2009). African-American respondents were less positive than white respondents about nursing home staff, trust, and quality (Bradley et al. 2004).

In case of dementia, age (OR: 0.96), being female (OR: 1.41), and marital status (OR: 0.53) significantly affected the intention of Japanese-Americans to use nursing home care (McCormick et al. 2002). In case of Alzheimer’s disease, institutional care was preferred by respondents with a higher education, better cognitive status, greater number of illnesses, and not wanting to become a burden on family (Werner and Segel-Karpas 2016). For a hypothetical stroke, independent decision-making style (OR: 7.96) increased the likelihood of choosing a mixed care arrangement instead of informal care. An independent decision-making style (OR: 9.83), having health insurance coverage (OR: 12.72), and greater IADL limitation (OR: 3.53) increased the odds of relying on all formal care (Min 2005).

### Block 3: Preferences for the design and structure of specific LTC services

#### Home-based and community-based care

Six studies investigated people’s preferences for home-based services (package A vs. B) for care-dependent people by means of a DCE (Chester et al. 2017, Kaambwa et al. 2015, Kampanellou et al. 2019, Lehnert et al. 2018, Walsh et al. 2020, Chester et al. 2018). While Chester et al. (2017) and Kampanellou et al. (2019) asked British caregivers to people with dementia (PWD) for their appraisal, Kaambwa et al. (2015) and Chester et al. (2018) questioned care-dependent people as well as informal caregivers. Lehnert et al. (2018) and Walsh et al. (2020) surveyed a sample of the general population. Less rotation in the number of caregivers per month were valued as crucial by respondents in the DCE of Chester et al. (2017) and Lehnert et al. (2018). In the DCE by Lehnert et al., respondents were willing to pay up to 213.86€ per month for a regular caregiver. When it comes to dementia care, several studies have found specialized training and communication skills to be the most important attributes for respondents. In a second DCE by Chester et al. (2018), “support with personal feelings and concerns provided by a trained counsellor at home” (Coeff: 0.676, WTP: £31) and “information on coping with dementia provided by an experienced worker at home” (Coeff: 0.592, WTP: £27) were valued the highest. In the study by Walsh et al. (2020) “personalized communication with the person with dementia” (Coeff: 0.54, WTP: €135.45) was found to be one of the most important determinants of person-oriented home-care services for PWD. Guzman et al. (2019) investigated the type of communication skills. It found that non-verbal communication (eye contact, tone of voice, body language) had the greatest significance to the respondents. Especially implementing interventions or procedures promptly and completely (Coeff: 6.763) and listening attentively to verbalization of patients (Coeff: 4.732) was most important.

Loh and Shapiro (2013) assessed the willingness to pay for home- and community-based services (HCBS). On average, respondents were willing to pay up to $933.32 for HCBS per months. WTP varied across different HCBS programs, with the highest WTP of $1776.61 documented for Alzheimer Disease Initiative program (Loh and Shapiro 2013). Lehnert et al. (2018) found respondents of the German DCE were willing to pay up to €233.71 per month and up to €429.10 per month for high quality care and very high quality of care, respectively. Walsh et al. (2019) found respondents were willing to pay up to €154.18 for “20 h per week of publicly funded care hours” and up to €139.64 for “high flexibility of service provision.” Furthermore, Kampanellou et al. (2019) found that respondents ranked the attributes “respite care for you is available regularly to fit your needs” (Coeff: 1.292; WTP: £235) and “home care such as personal care and cleaning is provided regularly for as long as needed” (Coeff: 0.933; WTP: £170) to be the most important.

In the study by Kaambwa et al. (2015) the most preferred community aged care (CDC) package that was chosen across all subgroups with a probability of 0.124 was the one with multiple service providers as well as family members to provide day-to-day services. The individual (client) was responsible for managing funds. Another Chinese study by Xiang et al. (2019) found the provision of health care services the most important, of which regular health examination (mean priority: 1.51) and health counselling (mean priority: 2.46) were the most important. Culture-related activities were judged as the least important by the respondents, although it was deemed slightly more crucial by the male respondents compared to the women. Respondents’ education levels also influenced their answers. Respondents with a higher educational level placed greater importance on daily life assistance.

### Long-term care facilities

Three studies examined people’s preferences for LTC facilities or nursing homes by means of a DCE (Milte et al. 2018b; Sawamura et al. 2015; Song et al. 2020). In an Australian study by Milte et al. 2018b, residents of nursing homes as well as family members as proxies were surveyed; the study focused on food preferences. The most important attribute of respondents was the taste of the food, whereby it was judged that it is crucial for the food that is provided to have an excellent taste (Coeff: 0.558, WTP: $24 per week). In the Japanese DCE by Sawamura et al. (2015), respondents had to choose between facility A and B for two different patient profiles, one with dementia and the other one with a fracture. Respondents valued the facility where relocation was not required as the highest even when the health of the patient deteriorates (*dementia* coeff: 1.67, *fracture* coeff: 1.36). Respondents with personal caregiving experience showed significantly greater preference for the availability of individual choice of daily schedule and meals. In the Chinese study by Song et al. (2020), older adults with the intention or willingness to live in a nursing home were asked to choose either between nursing homes A and B or neither of the options. The most important attributes for respondents were “location” and “care service”. The respondents preferred inner suburbs and regarded good care service as important.

In a large study by Roberts and Saliba (2019), assessment data of nursing homes was used to rank 16 daily care and activity services on a Likert scale from one to five. The latent class model showed that preferences could be grouped into four classes, namely “important” (38.3%), “activity” (27.1%), “care” (24.1%), and “unimportant” (10.4%). Race, ethnicity, cognition, and depression were found to be predicting values to determine the group to which the respondents were most likely to belong. African-American race and Hispanic ethnicity were predictors of membership to the first group, which ranked almost all care and activity preferences as important. Cognitive impairment and depression were predictive factors of belonging to the fourth group, in which respondents ranked more than half of daily care and activity preferences as unimportant.

In a survey study by Abbott et al. (2018), changes in preferences of 255 nursing home residents were examined over a period of three months. Sixteen of 72 preferences were rated as very or somewhat important by 90% or more of the residents. These preferences fell predominantly into the domain of self-dominion (*n *= 9) and enlisting others in care (*n* = 4). A total of 96.50% of respondents preferred having staff show respect to nursing home residents. When asked again after three months, the average agreement rate was 59% although 68 of 72 preferences had 70% or higher stability over the time period. Results reveal that residents who report high levels of importance at baseline are likely to report the same high preferences after the time period of three months. In a study by Przybyla et al. (2019), 214 senior Polish citizens were surveyed regarding their housing preferences. Willingness to move to housing options better tailored toward limited mobility was found in respondents living in larger cities. Based on the answers of the respondents, the preferred facilities at a senior housing estate included a 24 h medical care service (63%), a guarded estate (50%), having house cleaning services (48%), and a canteen (47%). A live-in caregiver was judged as important by less than 20% of respondents.

### Informal care

Mentzakis et al. (2011) conducted a DCE in Scotland with 209 informal caregivers to estimate monetary valuations for various informal care tasks. Initially, respondents were asked to choose between two hypothetical informal care situations, with the opportunity to opt out in a second step and let a person of their choice take over. A three-class model was fitted and illustrated preference heterogeneity between these three groups. Monetary compensation to the caregiver was judged as more important by younger respondents than older adults. For the first class, the attribute “household tasks” was the most important, with the lower the number of hours of household tasks per week being preferred (Coeff: -0.0109). For the second and third class, “personal care” was the most important attribute, where a lower number of hours of personal care per week was preferred (Coeff: -0.0232 and -0.0704 respectively). Willingness to accept values were additionally calculated. For class 1, the only statistically significant value was for household tasks being valued £0.6 per hour. For class 2, a per hour value of £0.38 for personal care was estimated.

Several studies tried to explore the value of informal care by estimating the willingness-to-pay (WTP) for a reduction in informal caregiving time through contingent valuation method (Gervès et al. 2013; Gustavsson et al. 2010; Fu et al. 2019; Liu et al. 2020; König and Wettstein 2002). Three of these studies (Gustavsson et al. 2010; Gervès et al. 2013; König and Wettstein 2002) focused on informal care for Alzheimer’s disease or other mental disorders. Gervès et al. (2013) surveyed French informal caregivers of elderly care recipients with cognitive impairments. The authors found that negative influences on caregiver’s morale was associated with the ability of respondents to estimate a WTP value for a 1 h reduction in care time. About 45% of the respondents were, however, unable to estimate a WTP value; 19% were willing to pay ≤ €13, 23% between > €13 and ≤ €18 and 13% were willing to pay more than €18 to be replaced for one hour. Gustavsson et al. (2010) surveyed a total of 517 informal caregivers of elderly care recipients in four countries. Mean WTP was calculated for a 1 h reduction in care need per day. For the UK, Spain, Sweden, and the US, the estimated values were £105, £121, £59 and £144 per month, respectively. In a Swiss study by König and Wellstein (2002), 109 pairs of informal caregivers and PWD were interviewed. On average, informal caregivers of the sample were willing to pay 57,500 CHF (US$ 38,000) for the complete elimination of burden and around SRF 2,200 (US$ 1,500) per year for a reduction of self-rated burden from moderate to low. For a hypothetical cure, caregivers were willing to pay up to 29% of their wealth and up to 23% to prevent future worsening of the disease. In a Chinese study by Liu et al. (2020), WTP and WTA values for 1 h reduction or increase of the least preferred care tasks of 371 informal caregivers were estimated. The average WTP of the respondents for a 1 h reduction of the least-preferred care task per week was 25.31 CNY. The minimum WTA for having to provide another hour of their least-preferred care task per week was 38.66 CNY. In a study by Fu et al. (2019) the number of co-payment respondents were willing to pay for voucher schemes in Hong Kong was investigated. Older age, greater financial resources, and a positive attitude toward voucher schemes resulted in a higher WTP of respondents.

**Table 4:** Included studies using choice-based (trade-off) techniques (n = 28)

Study^1^	Country	Population	Study objective	Method, Choice Scenario	Attributes, Levels
(21) Block 4	UK	162 unpaid carers for people over 65 years	To estimate preference-based index values for a profile measure of the caring experience (the Carer Experience Scale)	**BWS** Informal caregiving scenarios	1. *Activities outside caring* Most, Some, Few 2. *Support from family and friends* A lot of, Some, Little 3. *Assistance from organizations and the government* A lot of, Some, Little 4. *Fulfilment from caring* Mostly, Sometimes, Rarely 5. *Control over caring* Most, Some, Few 6. *Getting on with person you care for* Mostly, Sometimes, Rarely
(10) Block 2	USA	200 African-American and 200 White respondents ≥ 65 years, who had been hospitalized in the last year	To examine empirical difference in intended use of LTC by African-American and white elderly; to assess how factors medicate the effect of race/ethnicity on intended use of informal LTC	**Vignette** Intended use of informal LTC in case of 2 hypothetical scenarios (patient outcomes)	1. *Severity of disability* Cognitive (problems recalling events and recognizing familiar surroundings and people), physical and cognitive (unable to take a bath or use the toilet by yourself and cognitive impairment)
(29) Block 2	USA	215 older adults with median age of 73 and 51 adult children	To contribute to an understanding of the basis upon which older people and their adult children make decisions about residential options	**Vignette** Suitability of LTC options for four hypothetical outcomes	1. *Functional status* A visiting nurse has assessed name’s physical and functional abilities. Name has no difficulty in climbing a flight of stairs. She can drive her car safely under any normal road and weather conditions. She does not have trouble doing light housework, A visiting nurse has assessed name’s physical and functional abilities. Name is able to climb a flight of stairs but must use the handrail. She can drive her car safely but only within town and during daytime hours. She has some trouble doing light housework by herself, A visiting nurse has assessed name’s physical and functional abilities. Name has difficulty in climbing one flight of stairs. She is not able to drive. She cannot do light housework by herself 2. *Social network strength* Name has many good friends who live in her neighborhood, Name knows only a few people in the neighborhood; most of her friends have died or moved away, Name’s best friend lives in the retirement community that she is considering 3. *Current housing characteristics* Name lives in a house that has many features that make it safe and attractive for an older person, Name lives in a house with features that make it challenging for an older person 4. *Retirement community quality* Name is considering moving into a luxury retirement community in her area, Name is considering a move to a popular retirement community 5. *Financial implications* Name’s financial planner has determined that her monthly spending money would increase by $194 if she moves to the retirement community. Name’s financial planner has determined that her monthly spending money would increase by $85 if she moves to the retirement community, Name’s financial planner has determined that her monthly spending money would decrease by $80 if she moves to the retirement community, Name’s financial planner has determined that her monthly spending money would decrease by $175 if she moves to the retirement community 6. *LTC arrangements* Stay at home, move to retirement community
(65) Block 2	Switzerland	3195 older adults living in Lausanne between 68 and 82 years old	To assess the impact of incontinence on LTC choices among community-dwelling older citizens	**Vignette** Suitability of LTC arrangements for hypothetical patient outcomes	1. *Diverse needs for LTC ordered by type of severity (influencing factor: incontinence)* Urinary incontinence, fecal incontinence, mixed (urinary and fecal) incontinence 2. *Long-term care arrangements* Home, sheltered house, nursing home
(38) Block 3	England	28 carers of PWD	To test the applicability of the DCE method to assess carers' preferences for different attributes of home care for PWD	**DCE** Hypothetical home care packages (A vs. B)	1. *Home care workers use life story or memory wallets* Not at all, To some extent, Fully 2. *There is a waiting list for this service* No waiting list, 5 weeks, 10 weeks 3. *Home care workers are available* Day time only Mon-Fri, Night time Mon-Fri also if required, Weekends also if required 4. *Respite opportunities for carers* Not provided, Limited respite services, Full respite service for weekends and longer periods 5. *The home care worker visiting* Can be a different person each time, Varies from time to time, Is the same person each time 6. *The cost of this service is* £140 per week, £170 per week, £200 per week 7. *Home care workers have additional training in dementia care* No training, Some training, Full training
(52) Block 3	England	44 PWD and 103 carers of PWD	To explore the relative value of attributes of home support in dementia from the perspective of both PWD and their carers in relation to the early stages of dementia using the DCE approach	**DCE** Hypothetical home care packages (A vs. B)	1. *Advice on the use of memory aids (e.g. calendars, wall clocks) is* Not available, Available at a clinic, Provided by a trained worker at home 2. *Information on coping with dementia is* Available in writing only on request, Available over the phone or internet when needed, Provided by an experience worker at home 3. *Opportunities for social and recreational activities (e.g. walks) are* Not provided, Available through outside organizations, Provided by a dedicated worker at home 4. *Relaxation therapy is* Not available, Available at a clinic by appointment, Provided when needed at home 5. *Support with personal feelings and concerns is* Not provided, Available through a helpline, Provided by a trained counsellor at home 6. *Health promotion advice is* Not provided, Available at a clinic by appointment, Provided regularly at home 7. *The cost of the service, to you, is* £15 per week, £30 per week, £44 per week
(40) Block 3	Philippines	238 Filipino elderly patients from home health care and community settings	To understand elderly patients' preferences on the type of nurse-patient interaction in home care and community settings	**Conjoint analysis** Type of nurse-patient interaction	1. *Type of care provider* Staff nurse, Student nurse, Caregiver 2. *Gender* Male, Female 3. *Verbal communication* Nurse greets patient or returns greeting verbally, Nurse exchanges friendly/light comments or jokes showing ease with patient, Nurse makes clarifications/asks follow-up questions to further appraise for patient’s disease condition, Nurse gives adequate explanations patiently, Nurse volunteers needed information about procedures, meds, etc., Nurse provides verbal reassurance, Commands patient to do her instruction in a stern or irritated manner 4. *Nonverbal communication* Nurse ignores patient questions or comment, Nurse listens attentively to verbalization of patients on feelings, health condition and personal/family info etc., Implements needed interventions/procedures promptly and competently, Nurse attends only to the IV and other routine procedure and ignores the patient
(39) Block 2	Ireland	102 older hospital patients (65–80 years old) who do not suffer from dementia or delirium	To find out how people would balance and trade-off the different factors and outcomes that might arise if they developed significant dementia	**Conjoint analysis** Different hypothetical outcome scenarios for an 85 year-old with Alzheimer's disease (patient profile)	1. *Place of residence* Home with support, nursing home 2. *Risk of harm* High (80% chance of a potentially harmful incident within a year), moderate (50% chance of a potentially harmful incident within a year), low (20% chance of a potentially harmful incident within a year) 3. *Burden ("difficulty and strain") on family* A lot, some, little 4. *Life expectancy* 1 year, 2 years, 3 years
(37) Block 2	USA	81 respondents at short-term risk of needing LTC, but not currently receiving LTC	To quantify LTC preferences between different delivery modes	**TTO** Choice for six health states of either 1) living a longer life (another 10 years) or 2) shorter life with or without a given disability health state	1. *Functional impairment (cannot do the following without assistance of at least 1 person)* Different ADL needs: bathing, dressing, toileting, transferring, continence, feeding 2. *Cognitive impairment* None, Mild to moderate dementia, Moderate to severe dementia 3. *LTC arrangement* Home care, Nursing home
(30) Block 3	Australia	87 consumers of community aged care services delivered by 5 Australian providers and 30 informal caregivers	To explore clients' preferences for a variety of Consumer directed care (CDC) attributes and identified factors that may influence these preferences; to improve future CDC models	**DCE** Hypothetical CDC packages (A vs. B)	1. *Choice of service provider(s)* Single service provider, Multiple service provider, Multiple service providers and other individuals including family 2. *Budget management* The individual (client), An informal carer, The service provider 3. *Saving unused funds* Save all unused funds, save half of unused funds, Not able to save unused funds 4. *Choice of support/care workers* All your support workers, Some of your support workers, None of your support workers 5. *Support worker flexibility* Fully flexible, Partly flexible, Inflexible 6. *Level of contact with service coordinator* High contact (monthly), Medium contact (every three months), Low contact (every six months)
(5) Block 2	USA	1002 older women with moderate to severe disability in Baltimore	To describe caregiving arrangements and explore preferences for caregiving arrangements of older disabled women	**Vignette (Ranking)** Suitability of LTC arrangements (ranking from best to worst) for hypothetical patient outcomes	1. *Severity of disability* Person who needs help with meals every day, and with shopping, housework and transportation, but can take care of basic needs like bathing and dressing; person who needs help with bathing, dressing, and moving around in their residence on a daily basis, in addition to meals and other chores; person who needs help with personal and household activities and also has Alzheimer’s Disease or dementia which will get worse as time goes by 2. *LTC arrangements* In their own home with help from family and friends, Living with an adult child, Living in an assisted facility or a continuing care residence, Living in a nursing home, Living in their own home with help from someone paid to come in
(61) Block 2	USA	1783 Medicare beneficiaries aged 65 and older	To examine variations in care preferences and the extent to which preferences are congruent with or match current or future care arrangements	**Vignette** Suitability of LTC arrangements for hypothetical patient outcome	1. *Severity of disability* “Imagine a person named Pat, who is 80 years old with health problems. Because of these problems, he/she needs someone to help with bathing, dressing and getting around inside. Please look at this card and tell me what would be best for Pat?” 2. *LTC arrangements* Living in their own home with help from friends and family, Living in their own home with help from someone paid to come in, Living with an adult child, Living in an assisted living facility or continuing care residence, Living in a nursing home
(41) Block 3	England	100 carers of people in later stage dementia	To examine the relative importance of different home support attributes from the perspective of carers of people with later-stage dementia	**DCE** Hypothetical home support packages (A vs. B) with a budget of £230 per week to pay for care	1. *General home care such as personal care and cleaning is* Available irregularly, Available regularly but for short periods, Provided regularly for as long as needed 2. *Information on coping with dementia is* Available in writing only on request, Available over the phone or internet when needed, Provided by an experiences worker at home 3. *Respite care for you is* Not available, Available only for emergencies/special events, Available regularly to fit your needs 4. *Aids and adaptations in the home (e.g. light timers) are* Not available, Available if requested, Available regularly to fit your needs 5. *Training on how to manage behavior and difficulties is* Not provided, Provided in a support group, Provided by a dedicated worker at home 6. *Emotional support to you is* Not provided, Available through a ‘helpline’, Provided regularly at home 7. *The cost of service, to you, is* £75 per week, £115 per week, £150 per week
(42) Block 3	Germany	1.209 randomly selected participants from the general population aged 45–64 years	To investigate preferences for home- and community-based long-term care services packages (HCBS)	**DCE** Hypothetical HCBS packages (A vs. B) for 1 patient profile	1. *Care time (per day)* 30 min/day, 60 min/day, 90 min/day, 120 min/day 2. *Service level (range of services offered by the HCBS provider)* Standard, Extended 3. *Quality of care* Very high, high, satisfactory, sufficient 4. *Caregiver (per month)* 1–2, 3–5, 6–8 5. *Co-Payment (per month)* €0, €300, €600, €900
(3) Block 2	USA	1244 older Japanese Americans and 1354 Caucasian Americans	To compare attitudes toward the use of LTC between the two population groups	**Vignette** Suitability of LTC arrangements for hypothetical patient outcomes	1. *Severity of disability* Permanently disabled by dementia, temporarily disabled by hip fracture 2. *LTC arrangements* Move to a nursing home, receive paid home health care, be cared for at home by a loved one
(23) Block 3	Scotland	209 carers	To value informal care tasks and model the relationship between time spent on formal and informal care	**DCE** Hypothetical informal caregiving situations (A vs. B) in a first step, opportunity to opt-out in a second step	1. *Personal care (hours per week)* 0, 15, 30, 50 2. *Supervising (hours per week)* 0, 15, 30, 50 3. *Household tasks (hours per week)* 0, 15, 30, 50 4. *Formal care (hours per week)* 0, 15, 30, 50 5. *Monetary compensation (per hour compensation to the caregiver)* £0, £4, £10, £17
(43) Block 4	Australia	126 residents of nursing homes and 416 family member proxies	To generate a scoring algorithm weighted on the preferences of consumers for assessing the quality of care in nursing homes in sex key domains (CCI-6D)	**DCE** Alternative nursing home scenarios (Aged care home 1 vs. Aged care home 2)	1. *How much time are care staff able to spend with me/my family member?* Always able to, Sometimes able to, Rarely able to 2. *Do the shared spaces of the aged care home as a whole make you/your family member feel ‘at home’?* Very, Sometimes, Rarely 3. *Does your own room her make you/your family member feel ‘at home’?* Very, Sometimes, Rarely 4. *Is there access to outside and gardens in this aged care home?* Always easily able to, Sometimes able to, Unable to easily 5. *How often does the facility offer me/my family member things to do that make me feel valued?* Often able to, Sometimes able to, Rarely or occasionally able to 6. *How flexible is the aged care home with the care routines (e.g. when you/your family member gets out of bed, shower, eat your meals)?* Very flexible, Sometimes flexible, Not much flexibility
(44) Block 3	Australia	43 residents of aged care homes and 78 family member proxies	To undertake a detailed analysis of the preferences for how food and the dining experience are provided within aged care homes; to elicit consumer preferences and their willingness to pay for food service in aged care homes	**DCE** Two kitchens that provide food differently in the residential aged care home (kitchen 1 vs. kitchen 2)	1. *How good is the taste of the food provided?* Not very, Satisfactory, Excellent 2. *How much choice do I have over serving size?* No choice, A little, A lot of choice 3. *When do I choose what I would like to eat?* No choice, The day before the meal, At the time of serving 4. *When do I eat my meal?* Anytime I like, Within a 1-2 h range, At a set time 5. *How visually appealing is the food?* Not very, Satisfactory, Excellent 6. *How much extra money would I need to pay?* $0 per week, $10 per week
(9) Block 2	USA	144 older Korean Americans	To examine preferences for LTC arrangements by Korean Americans	**Vignette** Suitability of LTC arrangements for hypothetical patient outcomes	1. *Severity of disability* Hip fracture, stroke 2. *LTC arrangements* Informal caregiver at informal care location, paid helper at informal care location, paid helper at formal care facilities
(19) Block 2	USA	89 Mexican–American and 30 non-Latino White elders	To examine caregiver preferences in the event of hip fracture between Mexican–American and non-Latino White elders	**Vignette** Suitability of LTC arrangements for hypothetical patient outcome	1. *Type of disability* Hip fracture 2. *LTC arrangements* Formal/professional helper, informal helper/caregiver
(24) Block 2	Netherlands	1082 participants from a subsample of the Dutch Survey Sampling International Panel (general population, 50–65 years old)	To elicit preferences in the general elderly population for LTC services for varying types of patients	**DCE** LTC scenarios (packages) for 4 groups of hypothetical patients (frail and demented elderly, with and without partner)	1. *Number of hours of care per week* 4 h, 8 h, 12 h, 16 h 2. *Organized social activities* Not available, 1 Half day per week, 2 Half days per week, 3 Half days per week 3. *Transportation service* Available, Not available 4. *Living situation* Living independently at home, Apartment building in the proximity of care, Sheltered accommodation, Elderly or nursing home 5. *Who provides care* Regular care provider, Varying care providers 6. *Individual preferences* Standardized care, The content of care is determined individually 7. *Coordinated care services delivery* Have to arrange little, Have to arrange a lot 8. *Punctuality* Max. 15 min waiting time, Max. 1 h waiting time, Max. 2 h waiting time, Max. 3 h waiting time 9. *Waiting list in months* Directly available, 4 Months, 8 Months, 12 Months 10.*Co-payment per week* No co-payment, 50 Euro, 100 Euro, 150 Euro
(31) Block 2	Ireland	97 hospital patients with a history of falls, fracture or osteoporosis (aged 70 years or older)	To investigate how older people at risk for hip fracture would balance and trade-off between different factors and outcomes that might arise after a hip fracture	**Conjoint analysis** Outcome scenarios for a patient with poor functional recovery after a hip fracture	1. *Discharge location* Home with support, Nursing home 2. *Likely fall risk* Yearly, Three per year, One per month 3. *Predicted life expectancy* 4 years, 2 years, 1 year 4. *Views of family* Agree with discharge location, Disagree with discharge location
(55) Block 2	Switzerland	2985 community-dwelling persons aged 68 years or older residing in a Swiss region	To collect the opinions of community-dwelling persons regarding LTC arrangements for a diversity of disability profiles	**Vignette** Suitability of LTC arrangements for hypothetical patient outcomes	1. *Severity of disability* No ADL disability, mild disability (IADL only), moderate ADL disability, severe ADL disability, urinary incontinence, urinary and fecal incontinence, no cognitive impairment, moderate cognitive impairment, severe cognitive impairment 2. *Living situation of hypothetical person* Living with or without able-bodied spouse 3. *LTC arrangements* Usual home, sheltered home, nursing home
(32) Block 3	Japan	371 adults aged 50–65 years old in 8 cities in Japan	To clarify the priorities of the functions of LTC facilities from the viewpoint of future beneficiaries	**DCE** Hypothetical LTC facilities (facility 1 vs. facility 2)	1. *Availability of individual choice of daily schedule and meals* Not available, Partially available, Entirely available 2. *Regular care staff* Not available, Available 3. *Room* Shared (2–4) room, Personal Room 4. *Main daily interaction* Mostly alone, Mostly with staff and other residents, Mostly with family and friends 5. *Relocation because of medical deterioration* Necessary, Unnecessary 6. *Waiting time* Over 1 year, Within 1 year, Immediate occupancy 7. *Distance from present residence* Forty minutes by car, Twenty minutes by car, Within walking distance 8. *Monthly fee* 100,000 yen, 250,000 yen, 400,000 yen
(64) Block 3	China	293 Shanghai residents with the intention/ willingness to live in a nursing home, but not yet entered into one	To reveal the elderly people’s preferences for nursing homes; to clarify reason for utilization imbalance and put forward the efficient strategies for planning practice	**DCE** Nursing home A vs. B (with option to opt-out)	1. *Location* Downtown, Inner suburbs, Outer suburbs 2. *Distance to home* 10 min, 20 min, 40 min, 60 min 3. *Air quality* Good, General 4. *Comprehensive hospital* 5 min, 15 min, 30 min, 45 min 5. *Metro station* Yes, No 6. *Care service* Good, General 7. *Monthly fee* CNY 1500, CNY 2000, CNY 2500, CNY 3000
(66) Block 3	Ireland	551 individuals over 18 years of age	To understand public preferences for personhood in home-care services for people with moderate dementia	**DCE** Home-care packages (A vs. B vs. Status quo)	1. *Communication* Standardized, Personalized 2. *Flexibility* Low, High 3. *Number of care hours* 10 h per week, 15 h per week, 20 h per week 4. *Co-payment* No co-payment, Means-test co-payment, Compulsory co-payment 5. *Additional taxation per year* €50, €100, €150, €200, €250
(35) Block 2	Israel	484 Jewish community-dwelling adults aged 45 and over	To study the willingness to use institutional care vs. home care in hypothetical situations of permanent disability and Alzheimer’s disease	**Vignette** Suitability of LTC arrangements for hypothetical patient outcomes	1. *Severity of disability* Permanently physically disabled, diagnosed with Alzheimer’s diseases 2. *LTC arrangements* Being cared for at home by family members, being cared for at home by paid caregivers, being cared for in sheltered housing by caregivers in a nursing ward, being cared for in a nursing home by professional caregivers
(17) Block 2	USA	420 disabled older women receiving informal care	To investigate LTC preferences across 3 hypothetical scenarios and over one year of time	**Vignette** Suitability of LTC arrangements for hypothetical patient outcomes	1. *Severity of disability* IADLs needs, ADL needs, dementia 2. *LTC arrangements* In their own home with help from friends and family, in their own home with help from someone paid to come in, living with an adult child, in an assisted living facility or a continuing care residence, in a nursing home

Note: ^1^This column includes the content-related classification of each study into one of the four main entities (“blocks”)

### Block 4: Impact of LTC services on care-related quality of life and caregiver’s outcomes

Different instruments have been developed to measure care-related quality of life and caregiver’s outcomes to be used in economic evaluations. This provides an indirect measure of what caregivers wish for their LTC situation to look like. One of these preference-based index instruments is the *Carer Experience Scale* that was designed to capture the caring experience and consists of six domains, each with three levels. Al-Janabi et al. (2011) conducted a BWS experiment to estimate index values for England. For the surveyed informal caregivers, being able to do most of the activities you want outside caring (Coeff: 4.41) and getting a lot of support from family and friends (Coeff: 4.08) were selected most often as best.

The *Consumer Choice Index–Six Dimension* (CCI-6D) instrument was developed in Australia to assess the quality of care in nursing homes. Milte et al. (2018a, b) generated a scoring algorithm for the instrument by means of a DCE with 126 residents of nursing homes and 416 family member proxies. Always having the room set up to make the resident feel “at home” was the most important for residents (Coeff: 0.616). While this was equally important to family members (Coeff: 0.623), the most important item for family members was that care staff are always able to spend time with their care-dependent family member (Coeff: 0.648). The authors recommend using the resident scoring algorithm, as they have live experience and their preferences showed greater consistency across answers (Milte et al. 2018a).

**Table 5:** Results of the quality appraisal using the PREFS Checklist (*n* = 68)

Study	Author, year	P	R	E	F	S	Sum score
(1)	König *and* Wettstein (2002)	✓		✓	✓	✓	4/5
(2)	Laditka et al. (2001)	✓		✓	✓	✓	4/5
(3)	McCormick et al. (2002)	✓		✓	✓	✓	4/5
(4)	Pinquart *and* Sörensen (2002)	✓		✓	✓	✓	4/5
(5)	Kasper et al. (2000)	✓		✓	✓	✓	4/5
(6)	Chan et al. (2000)				✓	✓	2/5
(7)	Eckert et al. (2004)	✓		✓	✓	✓	4/5
(8)	Kim *and* Kim (2004)			✓	✓	✓	3/5
(9)	Min (2005)	✓		✓	✓	✓	4/5
(10)	Bradley et al. (2004)			✓	✓	✓	3/5
(11)	Fisher (2003)	✓		✓	✓	✓	4/5
(12)	Wang et al. (2004)	✓		✓	✓	✓	4/5
(13)	Imamoğlu *and* Imamoğlu (2006)	✓		✓	✓	✓	4/5
(14)	Jang et al. (2008)	✓		✓	✓	✓	4/5
(15)	Chung et al. (2008)	✓		✓		✓	3/5
(16)	Kim *and* Choi (2008)	✓		✓	✓	✓	4/5
(17)	Wolff et al. (2008)	✓		✓		✓	3/5
(18)	Costa-Font et al. (2009)	✓		✓	✓	✓	4/5
(19)	Min *and* Barrio (2009)	✓		✓	✓	✓	4/5
(20)	Tang et al. (2009)			✓	✓	✓	3/5
(21)	Al-Janabi et al. (2011)	✓		✓		✓	3/5
(22)	Gustavsson et al. (2010)	✓		✓	✓	✓	4/5
(23)	Mentzakis, Ryan *and* McNamee (2011)	✓		✓	✓	✓	4/5
(24)	Nieboer, Koolman *and* Stolk (2010)	✓		✓	✓	✓	4/5
(25)	Chou (2010)	✓		✓	✓	✓	4/5
(26)	Gerves et al. (2013)	✓		✓	✓	✓	4/5
(27)	Loh *and* Shapiro (2013)	✓		✓	✓	✓	4/5
(28)	Spangenberg et al. (2012)	✓		✓	✓	✓	4/5
(29)	Caro et al. (2012)			✓	✓	✓	3/5
(30)	Kaambwa et al. (2015)	✓		✓	✓	✓	4/5
(31)	Robinson et al. (2015)	✓	✓	✓	✓	✓	5/5
(32)	Sawamura et al. (2015)	✓		✓	✓	✓	4/5
(33)	Li and Wang (2016)				✓		1/5
(34)	Fernandez-Carro (2016)	✓	✓	✓	✓	✓	5/5
(35)	Werner *and* Segel-Karpas (2016)	✓		✓	✓	✓	4/5
(36)	Iwasaki et al. (2016)	✓		✓	✓	✓	4/5
(37)	Guo et al. (2015)	✓		✓	✓	✓	4/5
(38)	Chester et al. (2017)	✓		✓	✓	✓	4/5
(39)	Fahey et al. (2017)	✓	✓	✓	✓		4/5
(40)	Guzman, Jaurigue *and* Jimenez (2019)	✓		✓		✓	3/5
(41)	Kampanellou et al. (2017)	✓		✓	✓	✓	4/5
(42)	Lehnert et al. (2018)	✓		✓	✓	✓	4/5
(43)	Milte et al. (2018a)	✓		✓	✓	✓	4/5
(44)	Milte et al. (2018b)	✓		✓	✓	✓	4/5
(45)	Fu et al. (2019)	✓		✓	✓	✓	4/5
(46)	Liu et al. (2019)	✓		✓	✓	✓	4/5
(47)	Rong et al. (2019)	✓		✓		✓	3/5
(48)	Zeng et al. (2019)			✓	✓	✓	3/5
(49)	Roberts *and* Saliba (2019)	✓		✓	✓		3/5
(50)	Hrast et al. (2019)	✓		✓		✓	3/5
(51)	Przybyla, Heldak *and* Kurtyka-Marcak (2019)	✓		✓	✓	✓	4/5
(52)	Chester et al. (2018)	✓		✓	✓	✓	4/5
(53)	Abbott et al. (2018)	✓		✓	✓	✓	4/5
(54)	Hajek et al. (2017)	✓		✓		✓	3/5
(55)	Santos-Eggiman and Meylan (2017)	✓	✓	✓	✓	✓	5/5
(56)	Qian et al. (2018)	✓		✓	✓	✓	4/5
(57)	Qian et al. (2017)	✓		✓	✓	✓	4/5
(58)	Zhang et al. (2017)			✓	✓	✓	3/5
(59)	Huang et al. (2018)	✓		✓	✓	✓	4/5
(60)	Fu et al. (2017)	✓		✓	✓	✓	4/5
(61)	Kasper et al. (2019)	✓		✓	✓	✓	4/5
(62)	zeng et al. (2019)			✓	✓	✓	3/5
(63)	Liu et al. (2020)	✓		✓	✓	✓	4/5
(64)	Song et al. (2020)	✓		✓		✓	3/5
(65)	Carvalho et al. (2020)	✓		✓	✓	✓	4/5
(66)	Walsh et al. (2020)	✓		✓	✓	✓	4/5
(67)	Wei *and* Zhang (2020)	✓			✓	✓	3/5
(68)	Dong et al. (2020)	✓		✓	✓	✓	4/5

*P* Purpose*, R* Respondents*, E* Explanation*, F* Findings*, S* Significance

## Discussion

The aim of this systematic review was to summarize and synthesize available evidence on LTC preferences of older adults in need of care. For this purpose, a wide range of studies applying SP methods to elicit preferences were systematically searched. Sixty-six international peer-reviewed studies were included and relevant results were extracted. While this review focused exclusively on quantitative SP methods, the heterogeneous results of the included studies reflect the complexity of LTC. Even when the same methodology was applied, the studies differed with regard to their research focus, study population, sample size, analysis model, and study design. Studies conducted in 19 different countries were included, with a majority of studies conducted in Asia dealt with rapidly aging populations. The overview of attributes and levels used in the choice-based elicitation techniques (DCE, BWS, CA) provide an important impression on how the studies operationalized LTC and measured related preferences, as the choice of attributes is usually substantiated by extensive literature reviews and/or qualitative interviews with the target population.

Irrespective of the heterogeneity of studies, some consistent findings emerged. When presented with a set of LTC options, the majority of study participants preferred to “age in place” and make use of home-based services or informal care by family members in case of becoming care-dependent. Particularly for short-term or mild-to-moderate care needs (e.g., hip fracture), they generally preferred informal caregiving with some professional assistance if needed. Remaining in one’s own home was linked with maintaining independence, autonomy, control, and their social contacts. With increasing care-dependency needs, a shift in the preferences of study participants across all studies could be noticed toward the exclusive use of formal LTC services or nursing home care. For dementia patients, especially, nursing home care or a regular formal care provider at home was deemed important by respondents. Nevertheless, nursing home care was associated by many with a loss of freedom, independence, and dignity but preferred by some to not impose on family members in case of a greater care-dependency. Next to the severity of care needs, a few other independent variables were shown to influence LTC preferences, while none showed consistent effects across all studies. Being female, married with children, and already living with a partner or their children increased the likelihood of preferring informal or home-based care. A higher income, higher educational level, being unmarried or widowed, poorer health status, and having provided informal care in the past increased the likelihood of choosing nursing home care in the future.

The choice-based elicitation techniques showed that for home-based services, quality of care and less rotation in the number of caregivers per month were deemed to be crucial. In the selection of attributes, home-based packages tailored to informal caregivers to PWD placed a larger focus on the availability of respite and relaxation opportunities as well as sufficient support and coping strategies to deal with the disease. Specialized training and communication skills as well as support with personal feelings were very important to informal caregivers. For the design of LTC facilities, location, care services, individual choice of daily schedule, and excellent taste of served food were found to be the most important attributes for respondents. Even when focusing on one LTC service, attributes and levels differed greatly between studies. This aspect makes comparability difficult, however is attributable to the fact that country-specific conditions have been integrated into the particular survey designs. Nevertheless, the cost of services in terms of a co-payment played an important role and was integrated as an attribute in each of the 12 DCE studies.

Additionally, several contingent valuation studies assessed the value of informal care by estimating the WTP for a reduction in informal caregiving time. A dependency of such a WTP value on the financial status of the caregiver can be assumed and has been shown across studies. However, the influence of the care recipient's status (e.g., his or her health status), cultural and social backgrounds or country-specific care situations, have not yet been investigated and compared sufficiently. Nevertheless, studies also show that it may be difficult for respondents to provide a direct WTP or WTA value. Thus, indirect forms of questioning (e.g., DCE) may be an appropriate alternative, as shown in the study by Mentzakis et al. (2011).

Among all the choice-based elicitation techniques, DCE was mostly applied, enabling not only a ranking of the importance of attributes but also an assessment of which trade-offs respondents were willing to make for certain attributes. The use of conjoint analysis, AHP, DCE, and BWS to elicit LTC preferences is a relatively recent development as the vast majority of these studies have been published between 2015 and 2020. A major concern in the design and implementation of a DCE or conjoint analysis design is its complexity, comprehensibility, and feasibility. The fact that in more than 50% of these studies, older adults in need of care or older hospital patients were surveyed, sometimes in addition to informal caregivers, shows that these types of stated preference methods are not only suitable for targeting the general population. Preference elicitation techniques in the field of older adult care have largely been cross-sectional, measuring preferences at a single point in time. Thus, little is known about the changes in preferences across time and generations. The two studies in this review examining changes in preferences have done so in a period of three months or one year. Although extremely resource-intensive, further longitudinal studies could help understand changes in preferences.

Other reviews have tried to synthesize evidence on LTC preferences of dementia patients (Lepper et al. 2020), institutionalization factors for entering a nursing home for older adults (Luppa et al. 2010) or instruments for measuring outcomes within aged care (Bulamu et al. 2015). Such reviews underline the purpose of trying to reduce the complexity within the field of LTC by attempting to systematically reveal and condense the heterogeneity of results and studies. This systematic review builds upon the recent scoping review by Lehnert et al. (2019) by additionally capturing the period from February 2016 to October 2020 and including preferences for nursing home as well as dementia care. We included a total of 34 additional studies from 2016 to October 2020. The inclusion of these recent studies showcase the very dynamic research interest in LTC preferences. Additionally, two-thirds of the included DCE and conjoint analyses were conducted in this period. Such elicitation techniques have considerably increased in the last few years as a way to quantify preferences (Soekhai et al. 2019).

### Implications for research and policy

In the design of LTC systems, the demand for preference data has increased over the last few years as a way to integrate people’s priorities, needs and expectations. National governments need to establish sustainable and affordable LTC systems to evolve with the on-going demographic developments. The heterogeneous methods and LTC operationalizations used in the included studies mirror the national LTC systems and their confrontation with LTC in general. Thus, comparing preferences across countries is onerous. National preference data should be consulted especially for improving policies and LTC structures. Against the background of changing social developments, studies are needed that investigate LTC preferences and needs from the perspective of care-dependent older adults as well as (potential) informal caregivers. Further research is needed on people’s willingness to care as well as their realistic capabilities to ease the immense physical and psychological strain most informal caregivers experience. As informal caregiving is still considered as an essential pillar of most LTC systems, willingness to care as an indicator of the informal care potential of each country is vital. Nevertheless, to avoid caregivers becoming the next generation of care-dependent people due to immense burden, LTC services should ideally be available to supplement informal care right from the beginning. Therefore, preference-based data on how LTC services such as home-based or nursing home care should look like is needed.

## Conclusion

Irrespective of the heterogeneity of the included studies, the majority of study participants preferred to “age in place” and make use of home-based services or informal care by family members in case of becoming care-dependent. With increasing severity of functional and especially cognitive impairment, preferences shifted toward an exclusive use of formal care services. Several influencing factors were investigated and reported that might explain such preferences; however, none showed consistent effects across all studies. The inclusion of preference data in the design of LTC systems can constitute an important part in finding sustainable and affordable LTC solutions that specifically support caregivers and mirror the needs and wishes of people in need of care. As shown by the rapid rise in published studies in recent years, the research interest in LTC preferences is consistently increasing. Future research should additionally investigate the changes in preferences across time and generations as well as research people’s willingness to care and their realistic capability to care next to other responsibilities such as occupation and children in the household.

## Data Availability

The data generated during the current study are all presented in the publication.
